# Magnetically assisted intraperitoneal drug delivery for cancer chemotherapy

**DOI:** 10.1080/10717544.2018.1455764

**Published:** 2018-03-28

**Authors:** Milad Shamsi, Amir Sedaghatkish, Morteza Dejam, Mohsen Saghafian, Mehdi Mohammadi, Amir Sanati-Nezhad

**Affiliations:** a Department of Mechanical and Manufacturing Engineering, BioMEMS and Bioinspired Microfluidic Laboratory, University of Calgary, Calgary, AB, Canada;; b Center for BioEngineering Research and Education, University of Calgary, Calgary, AB, Canada;; c Department of Mechanical Engineering, Isfahan University of Technology, Isfahan, Iran;; d Department of Petroleum Engineering, College of Engineering and Applied Science, University of Wyoming, Laramie, WY, USA

**Keywords:** Intraperitoneal drug delivery, magnetic drug targeting, computational and mathematical tumor modeling, drug penetration depth, interstitial hypertension, desmoplasia, tumor microenvironment

## Abstract

Intraperitoneal (IP) chemotherapy has revived hopes during the past few years for the management of peritoneal disseminations of digestive and gynecological cancers. Nevertheless, a poor drug penetration is one key drawback of IP chemotherapy since peritoneal neoplasms are notoriously resistant to drug penetration. Recent preclinical studies have focused on targeting the aberrant tumor microenvironment to improve intratumoral drug transport. However, tumor stroma targeting therapies have limited therapeutic windows and show variable outcomes across different cohort of patients. Therefore, the development of new strategies for improving the efficacy of IP chemotherapy is a certain need. In this work, we propose a new magnetically assisted strategy to elevate drug penetration into peritoneal tumor nodules and improve IP chemotherapy. A computational model was developed to assess the feasibility and predictability of the proposed active drug delivery method. The key tumor pathophysiology, including a spatially heterogeneous construct of leaky vasculature, nonfunctional lymphatics, and dense extracellular matrix (ECM), was reconstructed *in silico*. The transport of intraperitoneally injected magnetic nanoparticles (MNPs) inside tumors was simulated and compared with the transport of free cytotoxic agents. Our results on magnetically assisted delivery showed an order of magnitude increase in the final intratumoral concentration of drug-coated MNPs with respect to free cytotoxic agents. The intermediate MNPs with the radius range of 200–300 nm yield optimal magnetic drug targeting (MDT) performance in 5–10 mm tumors while the MDT performance remains essentially the same over a large particle radius range of 100–500 nm for a 1 mm radius small tumor. The success of MDT in larger tumors (5–10 mm in radius) was found to be markedly dependent on the choice of magnet strength and tumor-magnet distance while these two parameters were less of a concern in small tumors. We also validated *in silico* results against experimental results related to tumor interstitial hypertension, conventional IP chemoperfusion, and magnetically actuated movement of MNPs in excised tissue.

## 1. Introduction

Despite improvements in the treatment of metastatic cancer, treating patients with peritoneal carcinomatosis (PC) has remained a significant challenge (Ceelen & Levine, 2015). Systemic chemotherapy has shown a limited efficacy in patients with PC, and been traditionally regarded as a palliative therapy (Lambert, 2015). Recently, the application of regional intraperitoneal (IP) chemotherapy alongside cytoreductive surgery has shown promise to treat peritoneal malignancy (Montori et al., 2014; Sloothaak et al., 2014; Wright et al., 2015). IP chemoperfusion exposes peritoneal neoplasms to high concentrations of anticancer drugs by delivering copious amounts of cytotoxic agents to the peritoneal region while minimizing the risk of systemic toxicity (Lambert, 2015). Nevertheless, the efficacy of IP chemotherapy is still limited due to the low penetration depth of cytotoxic agents into the tumor tissue. Under poor penetration depth, only cancer cells at the tumor periphery are exposed to effective concentrations of cytotoxic agents, and therefore, the risk of PC recurrence remains inevitable as a result of heterogeneous drug distribution (Minchinton & Tannock, 2006).

During IP chemoperfusion, drug penetration into tumor nodules is expected to take place by convective and diffusive modes of interstitial transport (Dewhirst & Secomb, 2017). However, the unique pathophysiology of tumor, comprising nonfunctional lymphatics, a spatially heterogeneous construct of leaky vasculature, and a dense ECM structure, gives rise to a number of interstitial obstacles that hinders both the convective and diffusive mechanisms of drug penetration (Chauhan et al., 2011; Au et al., 2016). The stress generated as a result of tumor growth compresses lymphatic vessels at the center zone of the tumor rendering them nonfunctional (Padera et al., 2004; Jain et al., 2014). Also tumor vessels are notoriously leaky and discharge inordinately high values of transcapillary fluid into the interstitium (Heldin et al., 2004; Dewhirst & Secomb, 2017; Soleimani et al., 2018). Taken together, vessel leakiness and lack of functional lymphatics give rise to an interstitial fluid pressure (IFP) that uniformly increases throughout central regions of the tumor while it steeply declines at the tumor periphery (Heldin et al., 2004; Chauhan et al., 2011; Liu et al., 2011). As a result, the interstitial fluid remains stagnant at the tumor core while the interstitial fluid velocity (IFV) assumes non-zero values at the tumor periphery. The IFV at the tumor periphery is radially outward and abolishes the convection-mediated penetration of therapeutic agents. Hence, diffusion remains as the only viable mechanism for drug transport in and penetration into the tumor interstitium (Dewhirst & Secomb, 2017). However, intratumoral diffusive transport of chemotherapeutic agents is also compromised due to the presence of a dense ECM structure of many desmoplastic tumors (Miao et al., 2015; Mitchell et al., 2017). Under such conditions, it is not surprising that the penetration depth of intraperitoneally injected drugs would barely exceed a few milliliters (Witkamp et al., 2001; Ceelen & Flessner, 2010).

One way to improve drug penetration into many tumors, including peritoneal neoplasms, is to directly target microenvironmental features that give rise to transport barriers (Al-Abd et al., 2015; Khawar et al., 2015; Valente et al., 2017; Zhang et al., 2017; Modarres et al., 2018). In this regard, two recent studies combining antiangiogenic therapy with IP chemotherapy in mouse xenograft models of ovarian (Shah et al., 2009) and colorectal (Gremonprez et al., 2015) cancers reported the reduced IFP and improved penetration of antineoplastic agents topotecan and oxaliplatin, respectively. Nevertheless, experimental evidence also suggests that regulating the high IFP of a tumor may not be sufficient to improve the penetration of macromolecular agents into peritoneal malignancies. Flessner et al. (2005) reduced the IFP to zero by removing the tumor capsule of abdominally implanted xenografts in mice. Nevertheless, the IFP reduction *per se* did not significantly improve the penetration of trastuzumab into tumors. It was postulated that the ECM structure should also be considered as an important source of resistance to macromolecular transport (Flessner et al., 2005; Ceelen & Flessner, 2010). Consequently, to augment drug transport, the tumor ECM may also need to be modified alongside tumor vasculature either via enzymatic ablation of ECM constituents or targeted inhibition of ECM synthesis in the tumor microenvironment (Khawar et al., 2015; Miao et al., 2015). However, stroma targeting strategies are known to be intricately interdependent and have limited therapeutic windows (Au et al., 2016). Furthermore, judicial dosing of the vasculature and ECM targeting therapies has remained a challenge thus far (Jain, 2013; Nakai et al., 2013). Thus, it is imperative to seek less complex methods that can be utilized to enhance intratumoral drug distribution during IP chemotherapy.

Magnetic drug targeting (MDT) method has been examined to improve drug penetration during IP chemotherapy. Drug-coated MNPs such as superparamagnetic iron oxide nanoparticles (SPIONs) are actuated with an externally applied magnetic field and directed to the area of interest *in vivo* (Nacev et al., 2012). MDT has been around for almost twenty years since its first clinical trial (Lübbe et al., 1996) and it has progressed for developing various magnetic carriers (reviewed in Veiseh et al. (2010)). Meanwhile, numerous computational models of MDT have been developed to predict the transport of MNPs *in vivo* and identify the most impactful factors that regulate the efficacy of MDT. *In silico* models of MDT have mostly revolved around the intravascular transport of MNPs (David et al., 2011; Patel, 2012; Shaw et al., 2013) while some recent models have incorporated the extravascular transport into their simulations (Nacev et al., 2010, 2011a, 2011b; Nacev, 2013; Ne’mati et al., 2017). Notably, Nacev et al. (2010, 2011a) and Nacev (2013) devised a useful framework capable of predicting the distribution of MNPs in and around blood vessels using three non-dimensionless parameters that delineate the interplay among magnetic forces, blood viscous forces, and particle diffusion. The authors exhaustively explored the parameter space and identified three generic modes of MNP transport (namely magnetic force dominated, velocity dominated, and boundary layer formation) in and around blood vessels. Later, the model of Nacev et al. (2010, 2011a) was improved by incorporating the non-Newtonian properties of the blood (Ne’mati et al., 2017). Al-Jamal et al. (2016) compared the results of the same computational framework (Nacev et al., 2010, 2011a) against experimental data of CT26 tumor-bearing mice and corroborated the existence of velocity dominated behavior and boundary layer formation. They also used the model to extrapolate the preclinical data to clinical conditions considering the different physiological aspects held in humans. Nacev et al. (2011b) also addressed the transport of systemically injected MNPs in metastatic lesions. They elicited vessel architecture from two-dimensional (2D) histological sections of liver and assessed numerically whether magnetic forces can be exploited to achieve uniform nanoparticle distribution in the cancerous tissue. Their results showed about two-fold increase in time averaged intratumoral concentration of magnetically delivered nanoparticles with respect to conventionally delivered therapy.

Computational models have also addressed the transport and penetration of anticancer drugs within peritoneal tumors (Au et al., 2014; Steuperaert et al., 2017). Au et al. (2014) incorporated spatial heterogeneity of tumor properties (e.g. vascularity, cellularity, and hydraulic conductivity) into their model and validated the simulated drug spatiokinetics against experimental data. Most recently, Steuperaert et al. (2017) showed the role of interstitial hypertension in poor drug penetration. Smaller tumor nodules exhibited lower IFPs and correspondingly greater penetration depths. The authors also showed that decreasing the high IFP via normalization of leaky tumor vessels can increase the drug penetration depth up to 29% in small tumor nodules while it has a negligible impact on the penetration depth of large tumors. Given the inadequacy of vessel normalization therapy to increase penetration depth into large tumor nodules, alternative methods need to be applied to expose the bulk of the tumor to cytotoxic agents. While recent experimental evidence indicates that MDT could be used to traverse MNPs across a tissue (Kulkarni et al., 2015), MDT has traditionally been used to isolate MNPs against blood flow and concentrate cancer therapeutics to perivascular sites.

In this work for the first time, we evaluated the use of MDTs to facilitate interstitial drug transport within peritoneal neoplasms. An *in silico* model was developed to assess the capability of MDT method in surmounting interstitial barriers and improving the performance of antineoplastic drugs. The model combines the features of the tumor microenvironment, external magnetic stimulation, and particle penetrations into tumor nodules. A realistic tumor pathophysiology accounting for elevated interstitial pressure and dense ECM was reconstructed and the effect of MDT parameters (namely magnet strength, tumor-magnet distance, and the size of magnetic particles) on drug spatiokinetics was investigated. The results indicate that interstitial barriers significantly impede the penetration of free cytotoxic agents paclitaxel and cisplatin into tumors. Nevertheless, actuated by adequately strong magnetic forces, drug-coated MNPs could readily surmount interstitial hypertension, trespass the tumor boundary, and reach relatively high concentrations within tumors with the radius range of 1 mm <*R* < 10 mm. An optimal radius of 200 nm and 300 nm for MNPs was shown to be effective in magnetic delivery of drugs to the medium (*R* = 5 mm) and large (*R* = 10 mm) sized tumors, respectively. The performance of this strategy in a small tumor (*R* = 1 mm) was, however, shown to be minimally dependent on MNP size. Also the larger the tumor, the greater the impact of magnet strength and tumor-magnet distance on the success of MDT. The accuracy of numerical results was evaluated against several previous studies and the model exhibited satisfactory performance.

## 2. Materials and methods

**Figure 1.**
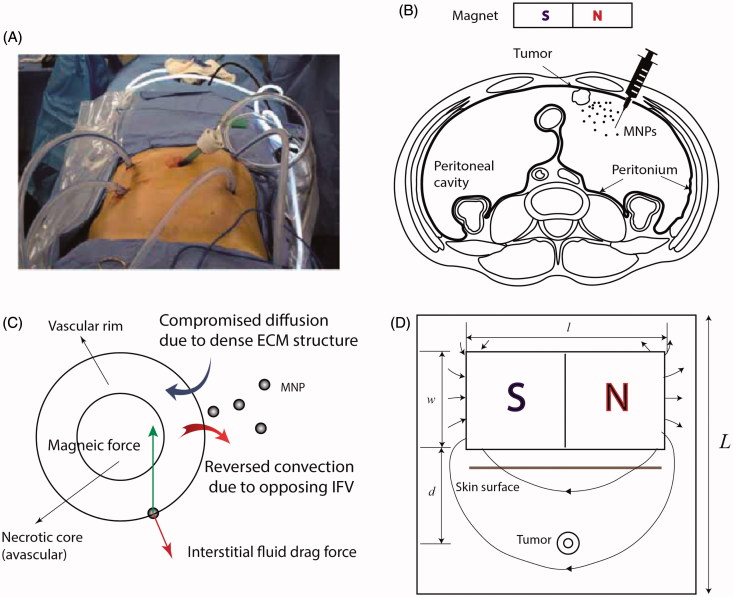
IP chemotherapy. (A) Clinical application of intraperitoneal (IP) chemotherapy (Wademan et al., 2012) (B) Schematic of the proposed magnetically assisted IP chemotherapy. Horizontal disposition of the peritoneum targeted with drug-loaded magnetic nanoparticles (MNPs). A permanent external magnet is utilized to impel MNPs across tumor nodules and surpass interstitial barriers. (C) Pathophysiology of tumors gives rise to opposing convective flows of the interstitial fluid at the tumor periphery which repel MNPs. Moreover, desmoplasia tends to hinder diffusive transport of MNPs. Magnetic forces can be applied to counteract these effects. (D) The geometry corresponding to the model of magnetically assisted IP drug delivery.

In clinical IP chemotherapy, chemotherapeutic agents are delivered to the target site through a heated pump in a continuous cycling within a course of 1–2 hrs (Figure 1(A)). The schematic of magnetically assisted IP drug delivery is illustrated in Figure 1(B). Drug-coated MNPs are administered intraperitoneally and an external permanent magnet is used to enhance drug penetration into the tumor. An idealized biologically relevant structure, consisting of an avascular necrotic core and a rim with leaky vasculature, was considered for the tumor (Soltani & Chen, 2011; Au et al., 2014; Steuperaert et al., 2017). Incorporation of a spatially heterogeneous construct of leaky vasculature along with the lack of functional lymphatics give rise to opposing IFV at the tumor boundary (Figure 1(C)). Moreover, we modeled compromised diffusion of MNPs as a result of a dense ECM structure. Figure 1(D) shows a schematic of the model geometry. A permanent magnet of length *l* and width *w* is placed at a distance of *d* from the tumor. The tumor is assumed to be circular with a radius equal to *R*. The radius of the necrotic core equals *R_n_*. Previous numerical models showed that the size of necrotic core has a trivial effect on drug penetration into tumors (Steuperaert et al., 2017). It is assumed that the size of necrotic core is half of the tumor size (i.e. *R_n_=R*/2). The values of all geometrical parameters are given in Table 1.

**Table. 1.** Model parameter values.

Parameter	Significance	Unit	Value	Ref.
*l*	Magnet length	cm	20	(Nacev et al., 2012)
*w*	Magnet width	cm	10	(Nacev et al., 2012)
*d*	Tumor-magnet distance	cm	5–20	–
*R*	Tumor radius	mm	1–10	(Steuperaert et al., 2017)
*K*	Hydraulic conductivity of the interstitium	m^2^ Pa^-1^ sec^-1^	3.0×10^-14^	(Baxter & Jain, 1989)
*L_P_*	Hydraulic conductivity of the microvascular	m Pa^-1^ sec^-1^	21×10^-12^	(Sefidgar et al., 2014)
*S/V*	Vasculature surface area per unit volume	m^−1^	2×10^4^	(Soltani & Chen, 2011)
*P_B_*	Vascular pressure	Pa	2.1×10^3^	(Soltani & Chen, 2011)
σ	Average osmotic reflection coefficient for plasma proteins	**–**	0.82	(Baxter & Jain, 1989)
π_*B*_	Microvessel osmotic pressure	Pa	2.7×10^3^	(Baxter & Jain, 1990)
π_*i*_	Interstitial osmotic pressure	Pa	2.0×10^3^	(Baxter & Jain, 1990)
χ	Magnetic susceptibility of magnetic particles	**–**	20	(Nacev et al., 2011a)
*B_rem_*	Remnant magnetic flux	*T*	0.5–2.5	(Ganguly et al., 2005; Nacev et al., 2011b)
*a_f_*	Radius of the tumor matrix fibers	nm	200	(Nacev, 2013)
ϕ	Volume fraction of tumor matrix fibers	**–**	0.66	(Levick, 1987)
*r_p_*	Pore radius of tumor vessels	nm	200	(Stylianopoulos & Jain, 2013)
δ	Vessel wall thickness	µm	5	(Stylianopoulos et al., 2013)
β	Drug elimination constant	s^−1^	7.32×10^-4^	(Steuperaert et al., 2017)
*IC*_50_	Half maximal inhibitory concentration	mol m^−3^	1.4×10^-6^ (Paclitaxel) 6.2×10^-3^ (Cisplatin)	(Steuperaert et al., 2017)

### 2.1. Governing equations

The *in silico* model of magnetically assisted IP chemotherapy consists of three main compartments accounting for the transport of interstitial fluid, magnetic forces, and transport of intraperitoneally injected MNPs in the cancerous tissue. The values of all model parameters are given in Table 1.

#### 2.1.1. Interstitial fluid

Darcy law in Eq. (1) is used to describe the relationship between the velocity and pressure of the interstitial fluid inside the tumor (Wu et al., 2014).

(1) $${\text{u}}_{\text{i}}=-K\nabla P_{i}$$

where *K* is hydraulic conductivity of the interstitium (m^2^ Pa^−1^ s^−1^), ***u_i_*** is interstitial fluid velocity (m s^−1^), and *P_i_* is pressure of the interstitial fluid (Pa). Moreover, the steady state continuity equation for the incompressible interstitial fluid is presented in Eq. (2) (Soltani & Chen, 2011).

(2) $$\nabla .{\text{u}}_{\text{i}}=\varphi_{B}-\varphi_{L}$$

where *φ_B_* and *φ_L_* are volumetric flow rates of plasma out of (into) vasculature (lymphatics) per unit volume of the tissue (s^−1^). *φ_L_* is set to zero due to the absence of functional lymphatics inside the tumor (Padera et al., 2004; Jain et al., 2014). Furthermore, the vascular contribution *φ_B_* is computed as Eq. (3) (Sefidgar et al., 2014; Soltani & Chen, 2011).

(3) $$\varphi_{B}=\frac{L_{P}S}{V}[P_{B}-P_{i}-\sigma_{s}(\pi_{B}-\pi_{i})]$$

where *L_P_* is hydraulic conductivity of the microvascular wall (m Pa^−1^ s^−1^) and *σ_s_* is average osmotic reflection coefficient for plasma proteins. Also *P_B_*, *P_i_*, *π_B_*, and *π_i_* denote vascular pressure, interstitial fluid pressure, microvessel osmotic pressure, and interstitial osmotic pressure, respectively. Moreover, *S/V* and *L_p_S/V* are known as vasculature surface area per unit volume and capillary filtration coefficient, respectively. Defining the effective pressure as *P_e_=P_B_-σ_s_(π_B_-π_i_)*, Eq. (3) is rewritten as Eq. (4).

(4) $$\varphi_{B}=\frac{L_{P}S}{V}(P_{e}-P_{i})$$

#### 2.1.2. Magnetic field

Because of the magneto-static nature of the problem, Maxwell-Ampere’s law is used to correlate the magnetic field ***H*** (A m^−1^) and the current density ***J*** (A m^−2^). Gauss’s law is used to model magnetic flux density ***B*** (V s m^−2^) (Eqs. (5) and (6)) (Haus & Melcher, 1989; Nacev et al., 2011a).

(5) ∇×H=J

(6) ∇.B=0

Given the presence of a permanent magnet, the current density ***J*** is set to zero. Furthermore, the constitutive equation ***B* ***= μ_0_****H*** is applied for air and tissue entities while the equation ***B* ***= μ_0_μ_r_****H* ***+**** B_rem_*** is used over the magnet domain (COMSOL, 2008). The magnetic permeability of the vacuum is set to μ_0_ = 4π × 10^−7 ^N A^−2^, the relative magnetic permeability of the magnet is set to μ_r_=1000, and the remnant magnetic flux is defined as ***B_rem_***. The magnetic force exerted on a single MNP in a magnetic field ***H*** is defined in Eq. (7) (Shapiro, 2009).

(7) $${\text{F}}_{\text{m}}=\frac{1}{2}V_{MNP}\frac{\mu_{0}\chi }{1+\chi /3}\nabla H^{2}$$

where *V_MNP_=4/3πa^3^* and χ denotes the volume and magnetic susceptibility of MNPs.

#### 2.1.3. Mass transport

*C_i_(****x****,t)* denotes the concentration (mol m^−3^) of drug-loaded MNPs at location ***x*** (m) and time *t* (s) within the tissue. The mass transport in the interstitium is modeled by the relevant partial differential equation of Eq. (8) (Baxter & Jain, 1989; Sefidgar et al., 2014)

(8) $$\frac{\partial C_{i}}{\partial t}+\nabla .(r_{F}\text{u}C_{i})=\nabla .(D_{e,t}\nabla C_{i})+\varphi_{s}$$

where *r_F_* is the retardation factor accounting for the hindrance of convective transport of drug nano-carriers due to the reflection by the porous tissue, ***u*** is the velocity of MNPs, *D_e,t_* is the effective diffusion coefficient of MNPs in the tissue (m^2^ s^−1^), and *φ_s_* is a source term. The velocity ***u*** is the sum of the local velocity of interstitial fluid ***u_i_*** and the so called equilibrium velocity ***u_e_*** with respect to surrounding interstitial fluid (***u* **=*** u_i_*** +***u_e_***) (Diver & Lubbe, 2007). We reach the equilibrium velocity ***u_e_*** when the Stokes drag force ***F_s_****= (6πaη)****u_e_*** equals the magnetic force ***F_s_***. Hence, ***u_e_*** is given by Eq. (9) (Nacev et al., 2011a; Nacev, 2013).

(9) $${\text{u}}_{\text{e}}=\frac{{\text{F}}_{\text{m}}}{6\pi a\eta }$$

where *η = 1.12 × 10^−3 ^*Pa s is the dynamic viscosity of the interstitial fluid (kg m^−1^ s^−1^). The effective diffusion coefficient of MNPs in the tissue is computed by the fiber matrix model (Fournier, 2012).

(10) $$\frac{D_{e,t}}{D_{B}}=\exp[-(1+\frac{a}{a_{f}})\phi^{1/2}]$$

where *a_f_* and *ϕ* denote the radius and volume fraction of tissue fibers, respectively. Brownian diffusion coefficient, defined as *D_B_* in Eq. (11), describes the diffusion of a particle of radius *a* in a fluid of absolute temperature *T* (K) and viscosity *η* (Gao et al., 2013).

(11) $$D_{B}=\frac{k_{B}T}{6\pi \eta a}$$

where *k_B_=1.381 × 10^−23^* m^2^ kg s^−2^ K^−1^ is the Boltzmann constant and *T* is taken equal to normal body temperature (310 K). The parameter *r_F_* is poorly known thus far (Diver & Lubbe, 2007). Some researchers have assumed that the retardation effect of the tissue is negligible and set *r_F_* to unity (Baxter & Jain, 1989, 1990, 1991) while others have scaled it with the reduced diffusion coefficient (Nacev et al., 2011a; Nacev, 2013; Ne’mati et al., 2017). We used the relation *r_F_=D_e,t_/D_b_* to compute the retardation factor. The source term *φ_s_* in Eq. (8) is computed by summing the vascular, lymphatic, and cellular contributions (Jain & Stylianopoulos, 2010; Sefidgar et al., 2014; Carlier et al., 2017).

(12) $$\varphi_{S}=[\frac{PS}{V}(C_{p}-C_{i})+\varphi_{B}(1-\sigma_{f})C_{p}]-\varphi_{L}C_{i}-\beta C_{i}$$

where the terms in the bracket constitute the vascular contribution and the last two terms are the lymphatic and cellular contributions, respectively. The parameter *C_p_* is concentration of MNPs in the plasma (mol m^−3^), *σ_f_* is osmotic reflection coefficient for MNPs, *P* is vascular permeability (m s^−1^), and *β* is drug elimination constant (*s^−^*^1^). Since MNPs are not delivered systematically, the plasma concentration *C_p_* is assumed to be zero (Steuperaert et al., 2017). The lymphatic term is also set to zero inside the tumor due to the absence of functional lymphatics (Padera et al., 2004; Jain et al., 2014). The vascular permeability *P* in Eq. (12) is computed as *P = D_e,e_/δ*, where *δ* is vessel wall thickness and *D_e,e_* is effective diffusion coefficient of MNPs in the endothelium (Kim et al., 1990), computed with the Renkine reduced diffusion coefficient model (Nacev et al., 2011a; Nacev, 2013).

(13) $$\frac{D_{e,e}}{D_{B}}={\left(1-\alpha \right)}^{2}(1-2.1044\alpha +2.089\alpha^{3}-0.948\alpha^{5})$$

where *r_p_* is the vessel pore radius and *α = a/r_p_*.

### 2.2. Initial and boundary conditions

For interstitial fluid transport, continuity of pressure and velocity on the interfaces of the tissue subdomains is imposed as the inner boundary conditions defined in Eq. (14). The interstitial pressure is set to a constant exterior pressure *P_ext_* on the outer boundary of the tissue as defined in Eqs. (15) and (16).

(14) $${-K\nabla P_{i}|}_{R_{n}^{-}}={-K\nabla P_{i}|}_{R_{n}^{+}}$$

(15) $${P_{i}|}_{R_{n}^{-}}={P_{i}|}_{R_{n}^{+}}$$

(16) $${P_{i}|}_{R}=P_{ext}$$

where *P_ext_* is set to the peritoneal pressure of −26.62 Pa measured in rat (Zhu et al., 1998). The magnetic insulation boundary condition is applied to the outer boundaries of the domain as prescribed by Ref. (COMSOL, 2008). For the mass transport of MNPs, we apply the continuity of concentration and mass flux on the inner boundary as defined in Eqs. (17) and (18) (Orlanski, 1976).

(17) $${\left(D_{e}\nabla C_{i}+r_{F}\text{u}C_{i}\right)|}_{R_{n}^{-}}={\left(D_{e}\nabla C_{i}+r_{F}\text{u}C_{i}\right)|}_{R_{n}^{+}}$$

(18) $${C_{i}|}_{R_{n}^{-}}={C_{i}|}_{R_{n}^{+}}$$

An inflow or outflow of MNPs can occur at the tumor boundary depending upon the local velocity ***u* **=*** u_i_*** +***u_e_***. We set an ‘inflow’ boundary condition on the regions with an inflow of MNPs into the tumor (COMSOL, 2012). An exterior concentration *C_ext_* is set to these inflow boundaries if it meets the condition defined in Eq. (19) (COMSOL, 2012).

(19) $$\begin{array}{ccc} C_{i}=C_{ext} & if & {\text{e}}_{\text{r}}.\text{u}<0 \end{array}$$

where ***e_r_*** is the unit radial vector at the tumor outer boundary. The chemoperfusion usually lasts for 60–120 min (Stewart et al., 2006). In our work, the duration of chemoperfusion is assumed to be 60 min, and the concentration of MNPs in the peritoneal region is assumed to be constant during the one-hour delivery. The exterior concentration *C_ext_* is set to 0.8 mol m^−3^ as prescribed in Ref. (Steuperaert et al., 2017). Alternatively, an ‘outflow’ boundary condition is applied on the regions of the tumor boundary with an outflow of MNPs (Nacev et al., 2011b; Nacev, 2013). According to the outflow boundary condition, the diffusive transport of MNPs is assumed to be negligible compared to the convective mode of mass transfer (Eq. (12)) (Nacev et al., 2011b; COMSOL, 2012; Nacev, 2013).

(20) $$\begin{array}{ccc} {\text{e}}_{\text{r}}.(-D_{i}\nabla C_{i})=0 & if & {\text{e}}_{\text{r}}.\text{u}\geq 0 \end{array}$$

Finally, the absence of magnetic MNPs in the tissue at *t = 0* refers to Eq. (21).

(21) $$C_{i}(\text{x},0)=0$$

### 2.3. Simulation method

Governing equations of the interstitial fluid transport, magnetic field, and mass transport were modeled and solved sequentially in COMSOL Multiphysics. The steady state distributions of the IFP, IFV, and magnetic field were used to solve the transient mass transport equation. The duration of IP chemotherapy was set to one hour and the time step of 45 s was used to solve the mass transport equation.

## 3. Results

The results of conventional IP chemotherapy of two widely used cytotoxic drugs, namely cisplatin and paclitaxel, are compared to the outcomes of magnetically assisted IP chemotherapy. The length and width of the magnet were set to *l* = 20 cm and *w* = 10 cm, respectively (Nacev et al., 2012). Three different parameters were defined to assess the performance of intraperitoneally injected drugs. First, the average interstitial concentration (*C_i,ave_*) is traced versus time. Second, the area under curve (*AUC*) of the *C_i,ave_* versus time curve is computed as an indicator of drug availability within the tumor. Third, the half width parameter *W_1/2_* is defined as the distance across the tumor surface where the concentration C_i_ equals the half exterior concentration *C_ext_* (Au et al., 2014). The half width *W_1/2_* represents drug penetration within the tumor. Additionally, the relative half width *W_1/2_*% is computed by dividing *W_1/2_* by the tumor radius *R* and used to compare drug penetration into tumors of different sizes.

### 3.1. Conventional IP drug delivery

**Figure 2.**
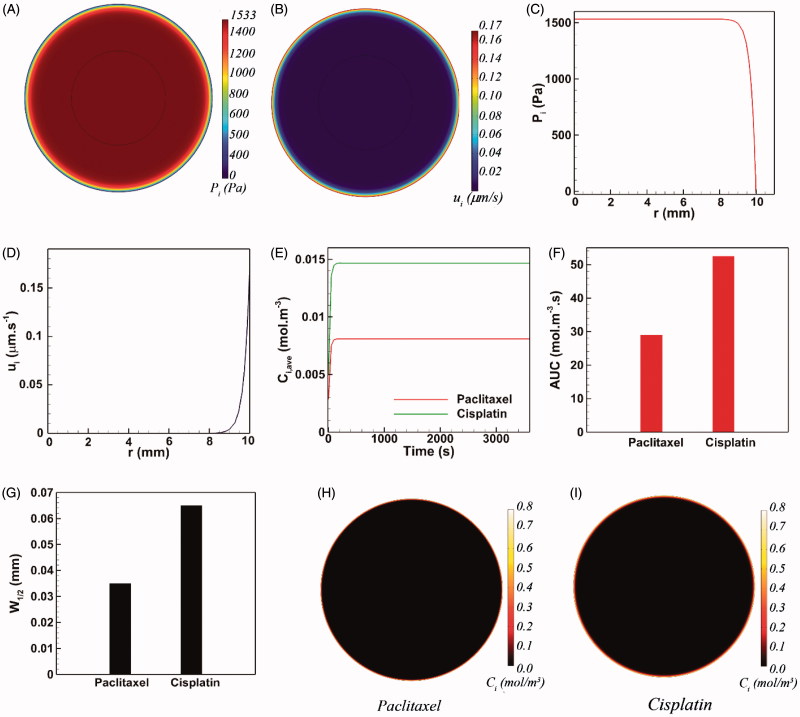
The conventional IP drug delivery. (A–D) The pathophysiology of tumor yields interstitial hypertension and opposing interstitial flow on the tumor surface as a result of the large interstitial pressure gradients near the tumor boundary, (E–F) The final intratumoral concentration of cisplatin is 1.75-folds greater than that of paclitaxel. Almost the same ratio holds between the *AUC* of these two cytotoxic agents, (G–I) Both paclitaxel and cisplatin show very limited penetration depths.

The dense ECM structure (modeled in Eq. (10)) and interstitial hypertension are the key parameters against drug penetration into the tumor tissue. Figure 2(A,B) shows the spatial distribution of the interstitial pressure *P_i_* and the magnitude of interstitial velocity ***u_i_***, respectively. A large central region with interstitial hypertension is generated within the tumor (Figure 2(A)) while the interstitial fluid remains stagnant over the same central region (Figure 2(B)). Figure 2(C) shows *P_i_* as a function of the tumor radius. The interstitial pressure reaches its maximum value (*P_i_* =1533 Pa) at the tumor center and is robustly maintained over most of the tumor radius until it starts declining steeply at the one tenth of the outer rim of the tumor. The magnitude of the interstitial fluid is directly proportional to the local pressure gradient (Eq. (1)). Hence, the magnitude of IFV is negligible for 0 < *r* < 9 mm (Figure 2(D)). However, the magnitude of ***u_i_*** drastically increases as a result of the steep pressure gradients at the vicinity of tumor periphery. Thus, the maximum value of the IFV (*u_i_* =0.17 µm s^−1^) occurs on the tumor surface (*r* = 10 mm) where it points radially outward and opposes the drug penetration into the tumor during IP chemotherapy. Therefore, it is expected that antineoplastic agents would be markedly hindered at the tumor boundary.

Cisplatin and paclitaxel are currently being used as antineoplastic agents for IP chemotherapy in clinics. IP chemotherapy was simulated by setting ***u_e_*** equal to zero, and thus setting ***u* ***=**** u_e_*** in Eq. (8). Moreover, the boundary conditions characterized by Eqs. (19) and (20) were changed to a simple Dirichlet boundary condition (*C_i_*|_R_= 0.8 mol m^−3^) to comply with the non-magnetically-assisted model (Au et al., 2014; Steuperaert et al., 2017). Figure 2(E) shows the average intratumoral concentration *C_i,ave_* of both cisplatin and paclitaxel versus time. *C_i,ave_* of both cytotoxic agents converges rapidly to definite values after about 200 sec and remains constant thereupon. At the end of the one-hour drug introduction, the average intratumoral concentration of cisplatin is 1.75 folds greater than that of paclitaxel. Moreover, cisplatin exhibits 1.8 folds greater *AUC* with respect to paclitaxel (Figure 2(F)). As expected, the opposing convective flow of the interstitial fluid near the tumor surface substantially reduces the penetration of cytotoxic drugs into the tumor. The half width *W_1/2_* of paclitaxel is only 35 µm (*W_1/2_*%=0.35%) (Figure 2(G)), and the half width of cisplatin equals 65 µm (*W_1/2_*%=0.65%). Figure 2(H,I) demonstrates that the penetration region of cytotoxic drugs is limited to a very thin outer rim of the tumor while the rest of the tumor remains untouched. Therefore, even though conventional IP chemotherapy can expose the exterior surface of the tumor to high concentrations of cytotoxic drugs, this delivery method is far from efficacious for large tumors due to a minimal drug penetration into the tumor core.

### 3.2. Magnetically assisted IP drug delivery: large tumor nodule

Intraperitoneal neoplasms range from micro-metastases (*R* < 1 mm) to large tumor nodules with *R* ∼ 10 mm. Given the poor penetration of cytotoxic drugs into tumors during conventional IP chemotherapy, larger tumors are more troublesome from a therapeutic point of view and pose a greater threat in terms of disease recurrence (Barakat et al., 2002; Ansaloni et al., 2015). In this section, we focus on a large tumor nodule with *R*= 10 mm. The results of drug penetration for the medium sized (*R* = 5 mm) and small (*R*= 1 mm) tumor nodules are discussed in **Section 3.3**. Three baseline values are set to three main MDT parameters (magnet strength *B_rem_* = 2.5 T, tumor-magnet distance *d* = 5 cm, and MNP size *a* = 100 nm). The influence of these parameters on MNP transport is studied by varying their values in a clinically meaningful range.

#### 3.2.1. Effect of magnet strength

**Figure 3.**
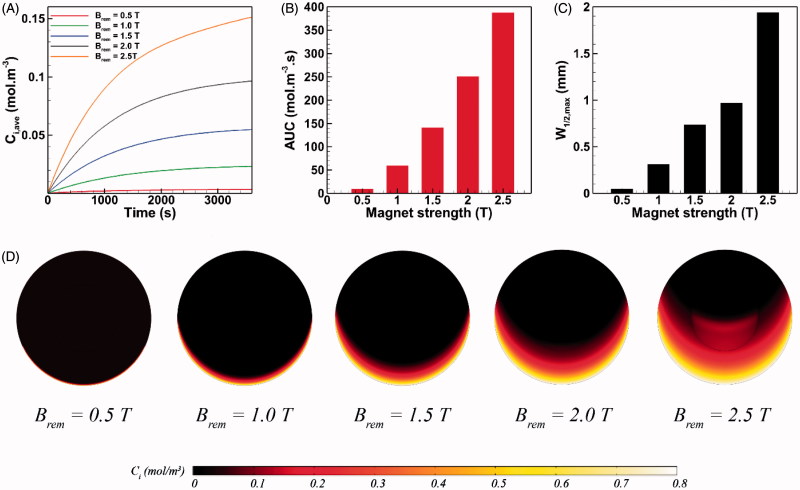
The effect of magnet strength on magnetically assisted IP drug delivery. (A–B) A 5-fold increase in the magnetic flux can significantly enhance C_i,ave_ and *AUC* of MNPs, (C) Maximum *W_1/2,max_* of 100 nm magnetic carriers is achieved under external magnetic stimulation with the strength of 2.5 T, (D) The upward directed magnetic force propels MNPs located on the lower half of the tumor against the interstitial fluid barrier and results in an enhanced penetration.

The strength of permanent magnet varies in the range of 0.5 T < *B_rem_*< 2.5 T (Nacev et al., 2012). The influence of magnet strength on the intratumoral transport of MNPs is presented in Figure 3. A 0.5 T permanent magnet applied to the drug-coated MNPs of 100 nm radius helps to reach an average drug concentration of *C_i,ave_* = 0.00362 mol m^−3^ within the tumor (Figure 3(A)), which is 55% and 75% less than achievable *C_i,ave_* values with paclitaxel and cisplatin, respectively (Figure 2(E)). This observation can be explained as the result of competition between two counteracting factors. First, compared to free cytotoxic agents, MNPs experience stronger transport retardation in the tissue as a result of their larger size. Second, MNPs benefit from impelling magnetic forces which helps them to surmount interstitial transport barriers. The former factor for 0.5 T magnet dominates the latter and therefore MNPs show a modest tissue transport. Nevertheless, with the aid of a 1.0 T magnet, the magnetic drug carriers with a radius of 100 nm can outperform free antineoplastic drugs and reach an average interstitial concentration of *C_i,ave_* = 0.0233 mol m^−3^ at *t* = 1 h, which is 1.66 times greater than the achievable *C_i,ave_* of cisplatin. Moreover, *C_i,ave_* of intraperitoneally injected MNPs at *t* = 1 h under a 2.5 T magnet can increase up to 41 times with respect to a 0.5 T magnet. Consequently, the *AUC* exhibits a 40-fold increase as a result of a 5-fold rise (from 0.5 T to 2.5 T) in the magnet strength (Figure 3(B)).

While the drug penetrates the tumor equally on every radial line in conventional IP chemotherapy (Figure 2(H,I)), the penetration pattern of MNPs does not exhibit circumferential uniformity in magnetically assisted delivery system (Figure 3(D)). Hence, the value of *W_1/2_* for MNPs varies depending upon their circumferential position in the tumor. Since the direction of magnetic force is upward on the tumor domain, *W_1/2_* reaches its maximum value on the vertical line passing the tumor center. This maximum half width in a magnetically assisted IP chemotherapy is denoted as *W_1/2_*_,_*_max_*. Also the relative maximum half width *W_1/2_*_,_*_max_* % is computed as the ratio of *W_1/2_*_,_*_max_* to the tumor radius *R*. Figure 3(C) shows the value of *W_1/2,max_* as a function of magnet strength for 100 nm MNPs. *W_1/2,max_* under external magnet strength of 0.5 T is about 50 µm which is 43% greater than the half width of free paclitaxel but 23% smaller than the achievable half width with free cisplatin (Figure 2(F)). Nevertheless, *W_1/2,max_* increases rapidly with the rise in magnet strength. For instance, *W_1/2,max_* exhibits a 5.2-fold increase and reaches 310 µm (*W_1/2,max_*% = 3.1%) by doubling the magnet strength to *B_rem_* = 1.0 T. The maximum value of *W_1/2,max_* equals 1.94 mm (W*_1/2,max_* % =19.4%) for magnetic carriers of *a* = 100 nm and under a 2.5 T magnet strength, which is about 55 times greater than the half width of paclitaxel and 30 times greater than the achievable *W_1/2_* with cisplatin. The enhancement of MNP penetration into the tumor is illustrated graphically in Figure 3(D). The upward directed magnetic force directs MNPs against the IFV on the lower half of the tumor while it adds up with the opposing IFV on the upper half of the tumor. As a result, MNPs penetrate the tumor on the lower half of the tumor. The greater the magnet strength, the deeper the penetration of MNPs.

#### 3.2.2. Effect of tumor-magnet distance

**Figure 4.**
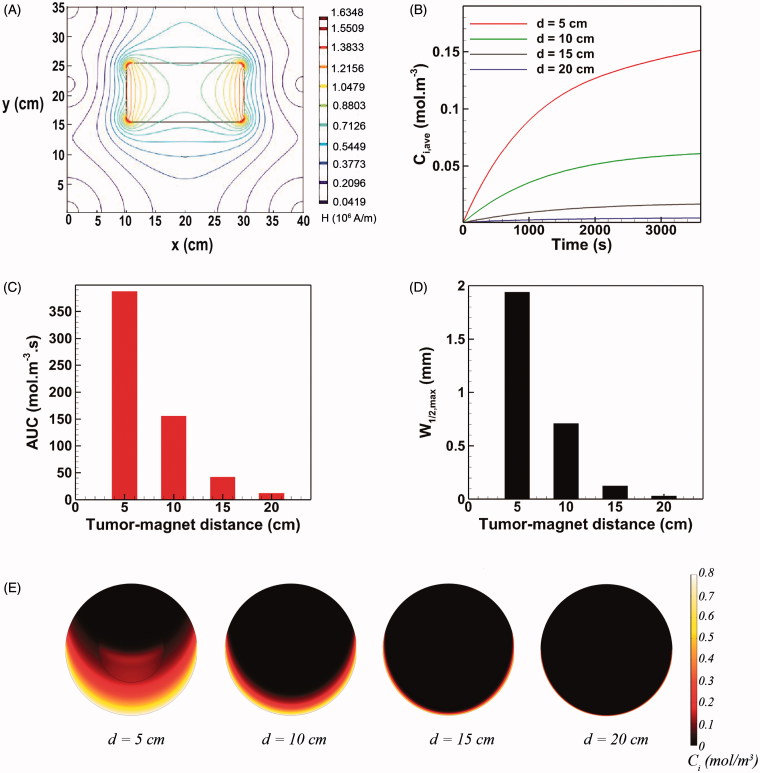
The effect of tumor-magnet distance on magnetically assisted IP drug delivery. (A) The gradients of magnetic field reduce rapidly by increasing the distance of tumor from the magnet. (B–D) The MDT performance parameters *C_i,ave_*, *AUC*, and *W_1/2,max_* strongly deteriorate as the tumor-magnet distance increases. (E) Interstitial retarding forces dominate magnetic forces for *d* > 10 cm and the drug penetration region remains limited to the lower half rim of the tumor.

The tumor-magnet distance can vary depending upon the location of tumor on the peritoneum (Figure 1(B)). Therefore, it is essential to assess the performance of magnetically assisted IP drug delivery for different distance of the tumor with respect to the magnet. According to Eq. (7), the magnetic force exerted on a single MNP is directly proportional to ∇*H*^2^. Hence, both magnitude and gradient of the magnetic field ***H*** contribute to the magnetic force. Figure 4(A) shows the spatial distribution of the magnetic field on the domain. The gradient of ***H*** is remarkably high near the magnet but rapidly decreases as moving away from the magnet. Accordingly, the MDT performance parameters namely *C_i,ave_*, *AUC*, and *W_1/2,max_* are expected to be strikingly impacted by the tumor-magnet distance.

At a tumor-magnet distance of *d* = 5 cm, the final mean concentration of MNPs equals 0.151 mol m^−3^ which is 10.3 times greater than the maximum achievable mean concentration with free cytotoxic agent cisplatin (Figure 4(B)). Accordingly, the achieved *AUC* shows a 7.4 time improvement with respect to conventional IP chemotherapy with cisplatin (Figure 4(C)). Moreover, as shown in Figure 4(D), the maximum half width *W_1/2,max_* for *d* = 5 cm equals 1.94 mm (*W_1/2,max_*%=19.4%) which is almost 30 times greater than that of the maximally penetrative free cytotoxic agent considered in this work (namely cisplatin). Increasing the tumor-magnet distance from 5 cm to 10 cm decreases *C_i,ave_* and *AUC* by 59% and 65%, respectively (Figure 4(B,C)). Moreover, as observed in Figure 4(D), a 63% loss in the maximum half width *W_1/2,max_*results as the tumor-magnet distance is doubled from *d* = 5 cm to *d* = 10 cm. Further increasing the distance to 15 cm decreased both *C*_i,ave_ and *AUC* by about 90% with respect to their baseline values (Figure 4(B,C)). Furthermore, the value of *W_1/2,max_* at *d* = 15 cm exhibits a 82% decrease compared to the maximum half width at *d* = 5 cm (Figure 4(D)). Finally, at *d* = 20 cm, all three performance parameters fall behind those of conventional IP chemotherapy and the MDT becomes completely ineffective.

Hence, a rectangular 20 cm ×10 cm permanent magnet with a strength of 2.5 T is not adequate for magnetically assisted IP chemotherapy of a large target tumor (*R* = 10 mm) located on very deep regions of the peritoneum. In qualitative agreement with our results, satisfactory MDT performance has been reported for depths not any greater than 10 cm in animal models (Goodwin et al., 1999). While a stronger magnet with *B_rem_*>2.5 T could be utilized to target deep seated tumors with *d* > 10 cm, we also note that the generated magnetic force depends on both the magnet strength and the gradient of the magnetic field (Eq. (7)). Hence, increasing the magnet strength alone may not suffice to favorably increase the magnetic force at distant points. An interesting approach that could be used to improve the reach of magnets while using the same magnet strengths is to reshape the magnetic field such that greater magnetic field gradients (and thus greater magnetic forces) are engendered at certain points of interest. This is usually done by juxtaposing an array of sub-magnets with different magnetization directions to form a so called Halbach magnet where the position of elements is optimized in order to maximize the magnetic force. For instance, the magnetic force generated by an optimized rectangular Halbach magnet (20 cm ×20 cm ×5 cm) consisting of 36 building blocks at a distance of 10 cm was reported to be 1.45 times greater than that of an ordinary permanent magnet possessing the same geometry and strength (Sarwar et al., 2012). Alternatively, the use of magnetizable implants (e.g. thin magnetizable wires or needles) or intra-corporeal magnets has been proposed to target deep seated tumors via MDT (Puri & Ganguly, 2014). However, the tractability of this approach may be limited by patient burden, physiological considerations, and post-implantation complications for the patient with peritoneal malignancy. Moreover, implant-assisted magnetic targeting seems to be mostly appropriate for primary tumors while peritoneal malignancy usually manifests as multi-sited disseminations. On the whole, tumor position on the peritoneum should be considered as one key player in magnetically assisted IP chemotherapy. MDT of tumors situated on deeper regions of the peritoneum is proved to be an open challenge that needs further investigation.

#### 3.2.3. Effect of MNP size

**Figure 5.**
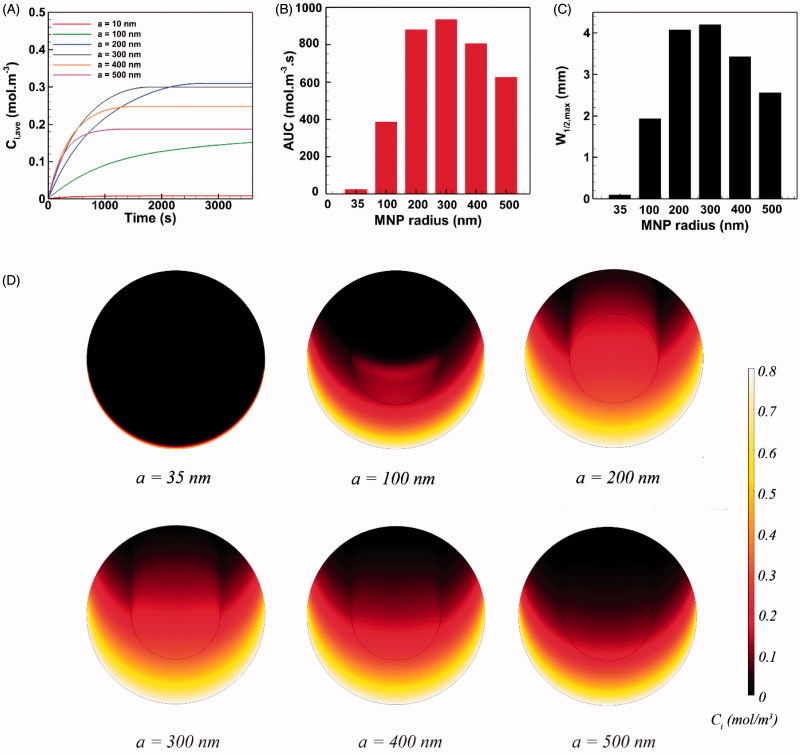
The effect of MNP size on the performance of magnetically assisted IP drug delivery. The MNP with radius of 300 nm exhibits the optimal performance and yields maximal intratumoral concentration, *AUC* and penetration into the tumor.

We examine the effect of MNP size in the radius range of 1–500 nm on drug delivery to peritoneal neoplasms. Based on Eq. (7), the magnetic force exerted on a single MNP is proportional to the particle size cubed. Decreasing the particle radius by a factor of 10 leads to a 1000-fold fall in the magnetic force exerted on the particle. Accordingly, we found that the magnetic force acting on particles with a radius of ∼10 nm is small and cannot effectively compete with opposing convective flows at the tumor periphery. Particularly, MNPs with the radius of below 35 nm were thoroughly repelled with opposing viscous forces and could not trespass the tumor boundary. Thus, the results of IP delivery were presented for MNPs larger than 35 nm (Figure 5).

The average intratumoral concentration of 35 nm MNPs equals 0.00813 mol m^−3^ at the end of the one-hour injection period. This value is almost the same *C_i,ave_* value obtained for free paclitaxel and is smaller than the *C_i,ave_* value of cisplatin. The *AUC* and *W_1/2,max_* values for 35 nm particles are similar to the values of free cytotoxic agents. Increasing the MNP radius up to around 200 nm improves the three parameters of drug delivery performance (*C_i,ave_, AUC*, and *W_1/2,max_*). Larger MNPs are more readily transported against interstitial barriers under a higher magnetic force. Also, larger MNPs are less cleared out from the tissue, particularly when the size of MNPs approaches the pore size of tumor vasculature *r_p_*. Nevertheless, increasing the MNP radius to around 300 nm declined the rate of increase in *AUC* and *W_1/2,max_* while the final *C_i,ave_* reduces. Intriguingly, further increasing the radius of magnetic carriers to 400 nm has an adverse effect on drug delivery performance and deteriorates the values of three parameters. This trend continues to exist by increasing the particle radius above 400 nm due to the increased tissue retardation against larger particles. It can be concluded that for particles larger than 300 nm, the increase in tissue retardation effect (due to the increased particle size) dominates the cumulative increase of magnetic force. Hence, the optimal size for MNPs is found to be 300 nm in radius, where *C_i,ave_* = 0.3 mol m^−3^ and *AUC* = 936 mol m^−3^, which is about 30 times greater than the values for free paclitaxel and 20 folds improvement with respect to free cisplatin. Moreover, the maximum half width (*W_1/2,max_* = 4.1 mm and *W_1/2,max_*% = 41%) improved significantly with respect to conventional IP drug delivery with free cytotoxic paclitaxel (*W_1/2,max_* = 35 μm and *W_1/2_*% = 0.35%) and cisplatin (*W_1/2_* = 65 μm and *W_1/2_*% = 0.65%).

### 3.3. Magnetically assisted IP drug delivery: medium and small tumors

Thus far, we focused on the improvement of drug delivery to a large tumor nodule (*R*= 10 mm) since experimental evidence indicates that larger tumors do not benefit from conventional IP chemotherapy (Barakat et al., 2002; Ansaloni et al., 2015). Nevertheless, we also simulated IP chemotherapy for medium sized (*R* = 5 mm) and small (*R* = 1 mm) tumor nodules (see Supplementary Information). Our results for conventional chemotherapy indicate that the value of the half width *W_1/2_* remains almost unchanged regardless of the tumor size (Figures S1(G) and S5(G)). Therefore, even though the IFV is less strong on the periphery of smaller tumors (Figure S5(D)), the diffusive transport is still negligible compared to opposing convective transport. As a result, the penetration depth is not subjected to changes for smaller tumors. Nevertheless, the relative half width *W_1/2_*% is higher for smaller tumors. Therefore, chemotherapeutics affects a greater fraction of the tumor body. Consequently, smaller tumors benefit from greater values of both *C_i,ave_* and *AUC* (Figures S1(E), S1(F), S5(E), and S5(F)).

Comparing Figures S2(C) and S6(C) with Figure S3(C) reveals that the smaller the tumor size, the higher the penetration of magnetically actuated nano-carriers (noted that the maximum achievable value of *W_1/2_* equals 2 mm for a small tumor of radius *R* = 1 mm). Smaller tumors exhibit higher values of *W_1/2_* under identical conditions of magnet strength, tumor-magnet distance, and MNP size. This is mainly because the opposing convective flow becomes weaker in smaller tumors, and magnetic forces can impel MNPs more easily against the IFV. Accordingly, given the same MDT variables, the smaller the tumor, the greater the values of *C_i,ave_* and *AUC*. Comparing Figures S2 and S6 with Figure 3 also reveals that the drug penetration into larger tumors requires a stronger magnetic strength. For instance, a 1.5 T magnet placed 5 cm away from a small tumor (*R* = 1 mm) increases *W_1/2_* up to 1.8 mm (*W_1/2.max_*% = 100%) while the same magnet can only achieve a half width of 0.74 mm (*W_1/2,max_*% = 7.4%) in a large tumor (*R* = 10 mm). Therefore, the magnet strength is less of a concern as the size of targeted intraperitoneal tumors reduces. Similarly, it is feasible to effectively target peritoneal surface malignancy of small tumors located at deeper regions. For instance, while a 2.5 T magnet, placed 10 cm away from a large tumor (*R* = 10 mm) can obtain a half width of 0.7 mm (*W_1/2,max_* = 7.1%), the half widths of 0.81 mm (*W_1/2,max_*% = 16.2%) and 1.9 mm (*W_1/2,max_*% = 100%) are achievable for a medium sized (*R* = 5 mm) and small (*R* = 1 mm) tumor nodules, respectively (compare Figures S3(C) and S7(C) with Figure S4).

Another interesting trend observed across different tumor sizes is the sensitivity of MDT performance to the choice of MNP size. Comparing Figure S4(C) with Figure S5(C) suggests that while the extent of drug penetration into the large tumor is less sensitive to the size of MNPs, the half width of a medium tumor markedly increases as the particle size increases. Nevertheless, as the size of tumor nodule further decreases, the performance of MDT is once again less dependent on the size of the selected magnetic nano-carrier. Therefore, an equal half width is achieved for a small tumor targeted with particles of radii ranging from 100 to 500 nm. Also the *C_i,ave_* and *AUC* are minimally sensitive to the choice of MNP size Figure S8(C).

### 3.4. Evaluation of model performance

**Figure 6.**
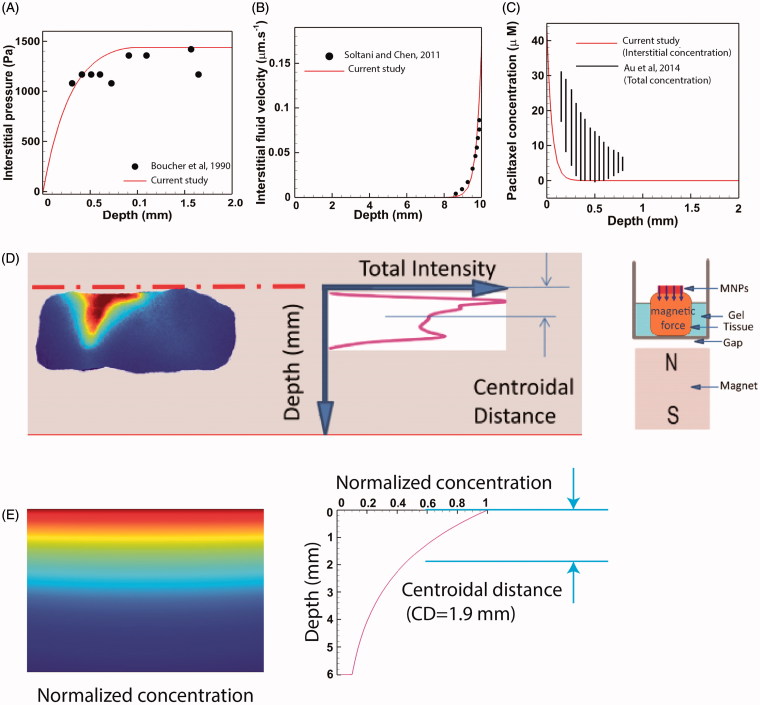
Validation of the model performance. (A) The theoretically computed IFP profile compared and validated against experimental data of Boucher et al. (1990), (B) The IFV-depth diagram of a large tumor (*R* = 10 mm) compared to the theoretical values of Soltani and Chen (2011), (C) The theoretical interstitial concentration of Paclitaxel compared to the total concentration (interstitial + internalized) of Paclitaxel obtained six-hours post IP chemotherapy in tumor bearing mice. Interstitial values are expectedly lower than total values but the theoretical and experimental diagrams share similar trends, (D) Schematic of the experimental methodology used by Kulkarni et al. (2015) to calculate the penetration depth of magnetically actuated MNPs inside an animal excised tissue, (E) *In silico* reproduction of experimental observations. The theoretical centroidal distance computed *in silico* (DC =1.9 mm) falls within the experimental range of 1.78–5.6 mm reported by Kulkarni et al. (2015).

The performance of our computational model was evaluated by comparing the results with previous experimental and numerical studies. Comparing the computed distribution of IFP on a tumor of radius *R*= 2 mm (Figure 6(A)) with the experimental data of Boucher et al. (1990) showed a good agreement between experimental and theoretical results. Also a remarkable agreement is reported when the IFV profile of a tumor of radius *R* = 10 mm was compared against theoretical values of Soltani and Chen (2011) (Figure 6(B)).

Following the approval of the performance of our flow solver, the results of our mass transport model were validated against previous studies. The result of mass transport model was first evaluated in absence of the magnetic field. Au et al. (2014) presented the experimental data of total (interstitial + internalized) concentration (*C_tot_*) of paclitaxel as a function of distance from tumor periphery for a tumor bearing mice six-hours post IP chemoperfusion. Similarly, we simulated conventional IP chemotherapy with Paclitaxel for a tumor of radius *R*= 2 mm over a six-hour period where the exterior concentration was set to *C_ext_* = 45 µM in order to comply with the values used by Au et al. (2014). Since our model accounts only for interstitial values of drug concentration (*C_i_*), we compared the theoretical values of *C_i_* against the experimental values of *C_tot_* (Figure 6(C)). Expectedly, the value of *C_i_* is less than *C_tot_* over the whole tumor radius. Nevertheless, the theoretically calculated *C_i_* shares the same trends as experimentally determined *C_tot_*. To further assess the results of conventional chemotherapy against previous studies, we define another drug delivery performance metric as the penetration depth (PD). PD has been defined as the distance across the tumor surface where the concentration *C_i_* equals the half maximal inhibitory concentration IC_50_ of the drug (Au et al., 2014). The values of IC_50_ for both Paclitaxel and Cisplatin are given in Table 1. The value of PD for a large tumor (*R* = 10 mm) is calculated to be 0.52 mm and 0.46 mm for paclitaxel and cisplatin, respectively. Our values fall in the ranges 0.54–0.75 mm and 0.36–0.49 mm reported for paclitaxel and cisplatin, respectively (Au et al., 2014). Moreover, our computed values are in compliance with the PD range of 0.41–0.56 mm reported for carboplatin (Ansaloni et al., 2015).

Finally, we seek to validate the results of our mass transfer model in the presence of magnetic forces. Kulkarni et al. (2015) investigated the transport of MNPs through an excised tissue and provided quantitative data for penetration of the particles. As shown in Figure 6(D), MNPs were placed on top of the excised tissue. A permanent magnet was located below the tissue to impel particles downward. The distribution of fluorescent particles was imaged after 45 min of proximity with the magnet. Images were processed and a curve characterizing fluorescent intensity versus tissue depth was produced. The centroid of this curve was used as an indicator of MNP penetration depth into the tissue. We incorporated these experimental data (the characteristic lengths of the problem, boundary conditions, magnet strength, duration of the experiment, and MNP size) into our *in silico* model and solved for the concentration field of MNPs of radius 50 nm. Figure 6(E) shows the interstitial concentration of MNPs after 45 min exposure to a magnetic field. As shown in the concentration-depth diagram, a centroidal distance of CD = 1.9 mm, computed in our simulation, falls within the range of 1.78–5.6 mm reported by Kulkarni et al. (2015), proving the performance of the mass transport solver in the presence of magnetic forces.

## 4. Discussions and conclusions

IP chemotherapy has been increasingly applied for the treatment of peritoneal surface malignancy. However, the poor drug penetration and consequent disease recurrence challenge the efficacy of this treatment. Experimental evidence suggests that the dense ECM of tumors along with interstitial hypertension should be held responsible for hindered drug penetration into peritoneal neoplasms (Flessner et al., 2005; Ceelen & Flessner, 2010). Recently, nano-sized drug carriers have been utilized in IP chemotherapy with the hope to improve intratumoral drug penetration and accumulation (Mirahmadi et al., 2010; Williamson et al., 2015). The rationale for the use of NPs in IP chemotherapy is to increase the residence time of antineoplastic agents in the peritoneal cavity, and thus to improve the chance for drug penetration into tumor nodules. In this regard, the larger the NP, the longer the retention time in the peritoneal cavity. Nevertheless, larger NPs are also less likely to penetrate deep into tumors. Therefore, a so called ‘size dilemma’ has been shown to exist in the application of nanomedicines to IP chemotherapy (Dakwar et al., 2017). We propose to resolve this size dilemma by exploiting magnetic nano-carriers actuated by external magnetic forces. In this method, one can benefit from an enhanced residence time of large NPs without losing the penetration depths.

We developed a computational model to predict the performance of the proposed magnetically assisted IP chemotherapy. Drug delivery barriers (dense ECM and opposing IFV) were reconstructed *in silico*, and the transport of magnetically driven MNPs inside a large tumor nodule of radius *R* = 10 mm was simulated. Conventional IP chemotherapy with free cytotoxic agents paclitaxel and cisplatin was simulated. Expectedly, the opposing IFV on the tumor’s outer rim thwarted drug penetration significantly and resulted in a very poor drug delivery performance (characterized by low values of *C_i,ave_*, *AUC*, and *W_1/2_*). The computational model was exploited to assess the impact of MDT parameters (magnet strength, tumor-magnet distance, and MNP size) on drug delivery to a large tumor. At its best, MDT performance parameters *C_i,ave_* and *AUC* of a large tumor were found to be 20 times greater than those of free cisplatin. The same performance parameters are almost 30 times greater than those of free paclitaxel. Furthermore, the optimal half width obtained by MDT was *W_1/2,max_* = 4.1 mm which is markedly greater than achievable half widths of paclitaxel (*W_1/2_* = 35 µm) and cisplatin (*W_1/2_* = 65 µm). We also simulated IP drug delivery to medium (*R* = 5 mm) and small (*R* = 1 mm) tumor nodules and showed that MDT yields better performance metrics as the tumor size becomes smaller. Moreover, MDT performance in the small tumor (*R* = 1 mm) was found to be less dependent on the MNP size.

A significant concern about nano-particle-based IP chemotherapy would be the particle deposition and concomitant toxicity in patient organs. In general, toxicity of MNPs has been stated to be a function of treatment dose, administration route, and particle features (e.g. composition, size, and surface properties) (Reddy et al., 2012). Brown Norway rats treated with IP administration (up to 3.7 mL/kg) of 10 nm iron oxide nanoparticles showed no remarkable morphological alterations in the spleen, lungs, or liver (Prodan et al., 2013). Moreover, while nude mice receiving IP injection (up to 90 mg Fe/kg body weight) of 10 and 25 nm iron oxide nanoparticles exhibited no sign of tissue injury (Pham et al., 2018), IP administration of iron oxide nanoparticles (20-40 mg/kg) in the size range of <50 nm led to severe hepatic and renal injuries in Kunming mice (Ma et al., 2012). As such, more systematic studies on organ specific bio-accumulation and toxicity of intraperitoneally administered MNPs are required to reach a general consensus on the safety and proper application of magnetically assisted IP chemotherapy.

The agreement between our results and previous experimental and numerical models suggests that our 2D tumor model can be adequately precise while reducing computational costs to minimum. The computational model developed here accounts for the spatial heterogeneity of vascularity and cellularity. Nevertheless, there are other microenvironmental properties (e.g. hydraulic conductivity of the microvascular wall and the interstitium) that may need to be accounted for. Moreover, we neglected the aggregation of MNPs in the tissue which may not be the case for all available magnetic nano-carriers. We also adopted a simplified model for cellular uptake of nanoparticles where the uptake term was assumed to be constant. Nevertheless, cellular uptake of NPs could vary depending upon the size of particles.

The ECM is a highly viscoelastic organ composed of fibrous proteins (namely collagen, elastin, fibronectin, and laminin) and proteoglycans (Frantz et al., 2010; Chauhan et al., 2011). Collectively, the ECM components constitute an intricately crafted dynamic structure that provide biomechanical and biochemical support to the cells. Moreover, the ECM acts as a selective barrier to particle transport in the extracellular environment based on the ‘size filtering’ and ‘interaction filtering’ mechanisms (Theocharis et al., 2016). The cutoff size of the ECM and particles as well as the particle–ECM chemical interactions are the main determinants of the extracellular particle diffusion (Lieleg & Ribbeck, 2011; Witten & Ribbeck, 2017). During tumorigenesis, various tumor and stromal elements act in concert to form an aberrant ECM exhibiting transformed matrix composition and topography (Theocharis et al., 2016). The transformed ECM has proven a significant barrier against drug penetration into peritoneal malignancy (Flessner et al., 2005; Choi et al., 2006). Both the size and interaction filtering effects may need to be incorporated into the mathematical model to faithfully predict the intratumoral transport of MNPs. Particularly, particle surface coating has shown to be a key player in regulating the interstitial transport of MNPs under applied magnetic fields (Kulkarni et al., 2015). Nevertheless, a comprehensive theory relating the properties of the particle (e.g. size and surface chemistry) and ECM (e.g. composition and topology) to the tissue transport of MNPs is missing. Therefore, we used a rather simple model that only accounts for the ECM steric barrier based on the particle size and volume ratio of the tissue fibers. Additionally, the effect of other factors such as recruited stromal cells on tissue resistance can complicate the issue. Yet, compared to experimental results, our reductionist model showed an acceptable performance in tissue penetration of magnetically actuated MNPs. Albeit, we are also aware that the 45-minute long *ex vivo* experiment used to validate our results may not be an adequately interrogative reference to judge the reliability of the computational model. Due to a paucity of relevant quantitative experimental data on MDT, a next meaningful step in our quest for enhanced drug delivery to peritoneal malignancy is to further evaluate the magnetically assisted IP chemotherapy under controlled conditions *in vivo*. In so doing, the necessity for the simultaneous incorporation of multi-faceted particle–ECM interactions (i.e. steric and non-steric filtering) could also be assessed and the model could be refined accordingly.

In summary, the computational model developed in this work was used to assess the utility of MDT in improving drug delivery to peritoneal malignancy. Our results predict that MDT can be exploited to enhance drug penetration into large tumor nodules which currently do not benefit from the locoregional IP treatment.

## Supplementary Material

IDRD_Amir_et_al_Supplemental_Content.docx
