# Supplementary material for: Magnetically assisted intraperitoneal drug delivery for cancer chemotherapy
Source: Drug Deliv. 2018 Mar 28;25(1):846–61. doi: 10.1080/10717544.2018.1455764 (PMC7011950; doi:10.1080/10717544.2018.1455764)
Supplement: IDRD_Amir_et_al_Supplemental_Content.docx [file IDRD_A_1455764_SM3534.docx]

**Magnetically assisted intraperitoneal drug delivery for cancer chemotherapy**

Milad Shamsi^1,2,3^, Amir Sedaghatkish^3^, Morteza Dejam^4^, Mohsen Saghafian^3^, Mehdi Mohammadi^1,2^, Amir Sanati-Nezhad^1,2*^

*^1^BioMEMS and Bioinspired Microfluidic Laboratory, Department of Mechanical and Manufacturing Engineering, University of Calgary, Calgary, AB T2N 2N1, Canada*

*^2^Center for BioEngineering Research and Education, University of Calgary, Calgary, AB T2N 1N4, Canada*

*^3^Department of Mechanical Engineering, Isfahan University of Technology, Isfahan 8415683111, Iran*

*^4^Department of Petroleum Engineering, College of Engineering and Applied Science, University of Wyoming, 1000 E. University Avenue, Laramie, Wyoming 82071–2000, USA*

**Supplemental Information**

The results of conventional and magnetically assisted Intraperitoneal (IP) chemotherapy of a medium (*R*= 5 mm) and small (*R* = 1 mm) tumor nodules is presented. The reader is referred to **Section 3.3** of the main text for the discussion on the results.

**S1. Conventional and magnetically assisted IP chemotherapy for a medium sized tumor (*R* = 5 mm)**

| 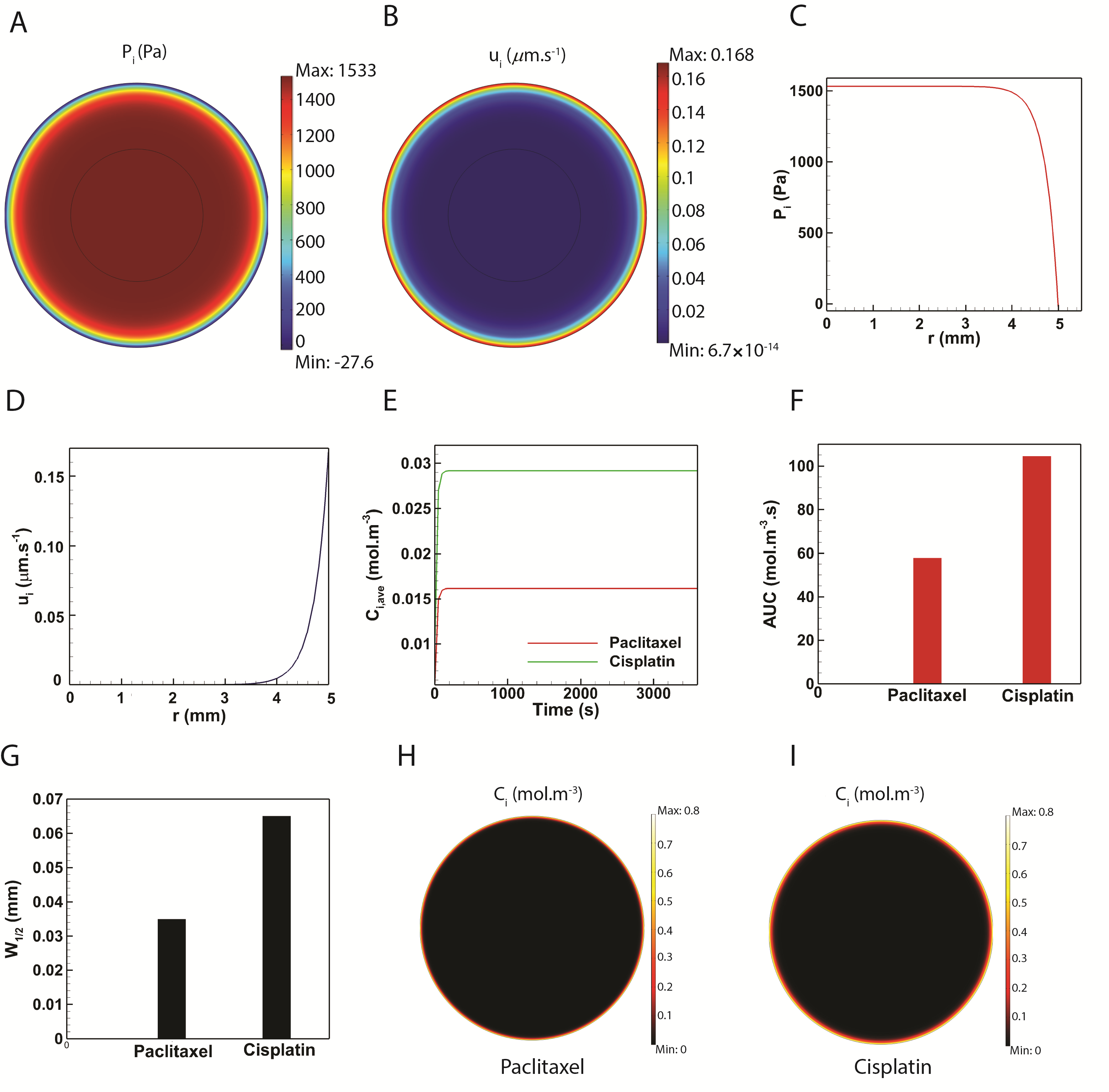 |
| --- |
| **Figure S1.** Conventional Intraperitoneal (IP) chemotherapy to a medium sized tumor nodule. (**A-D**) Maximum values of IFP and IFV remain almost unchanged with respect to those of the large tumor of radius 10 mm. (**E-F**) While the opposing convective forces remain almost the same with respect to those of a large tumor (*R*=10 mm), the values of *AUC* and final C_i,ave_ of a medium sized tumor are almost two times greater than those of a large tumor.(**G-I**) The value of the half width *W_1/2_* does not vary with respect to a large tumor (*R*=10 mm) while a larger percentage of the tumor bulk is covered with cytotoxic agents. |

| 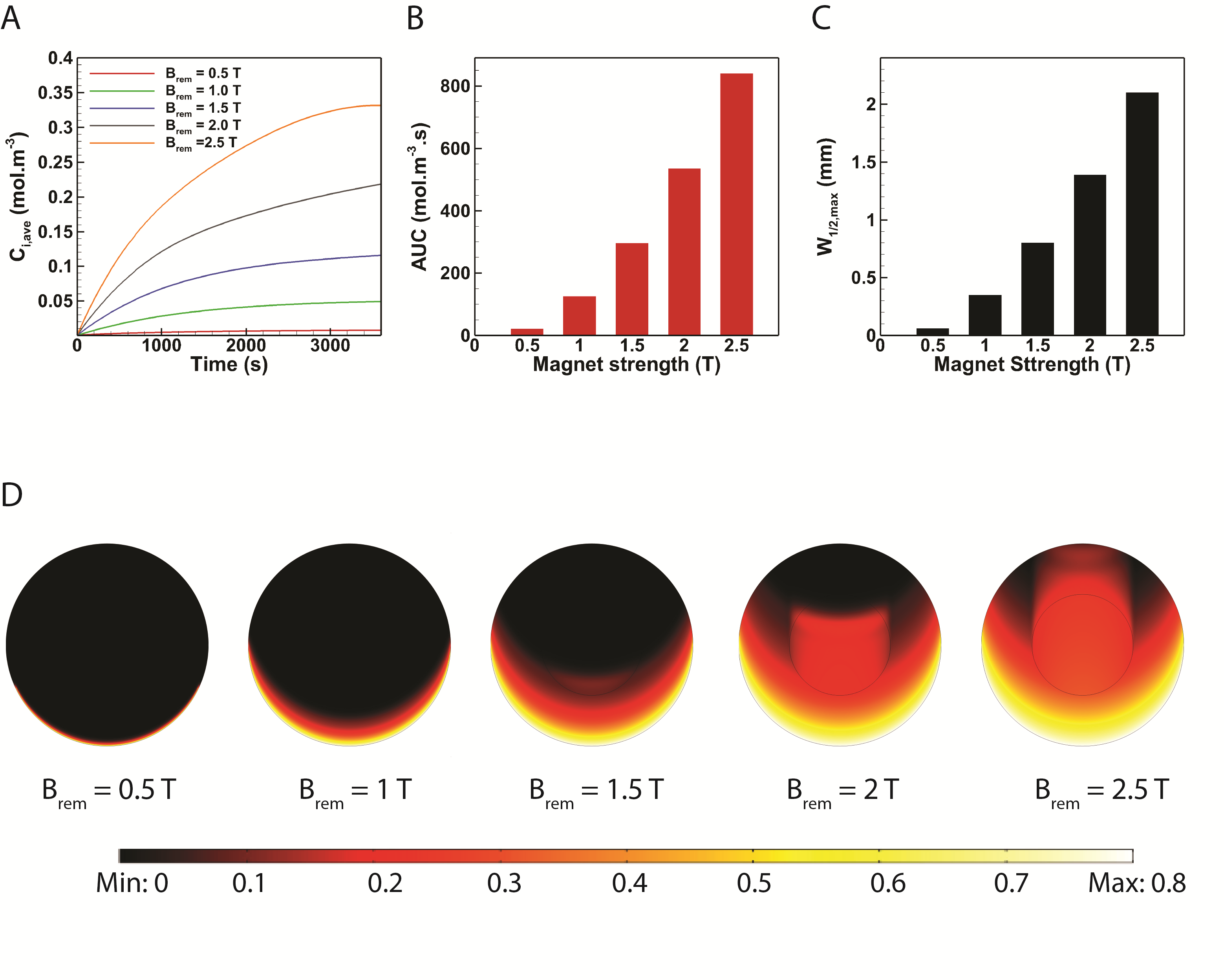 |
| --- |
| **Figure S2.** The effect of magnet strength on magnetically assisted IP drug delivery to a medium sized tumor nodule. (**A-B**) A five-fold increase in the magnet strength from *B_rem_*=0.5 T to *B_rem_*=2.5 T results in 40 times greater values of *C_i,ave_* and *AUC.* The medium and large tumors are equally sensitive to the choice of magnet strength. (**C**) Similarly, the maximum half width *W_1/2,max_* exhibits a 35-fold increase as a result of a 5-fold increase in the magnet strength, which is close to the 38-fold increase observed in a large tumor. (**D**) Intratumoral MNP distribution markedly improves by applying stronger magnets. |

| **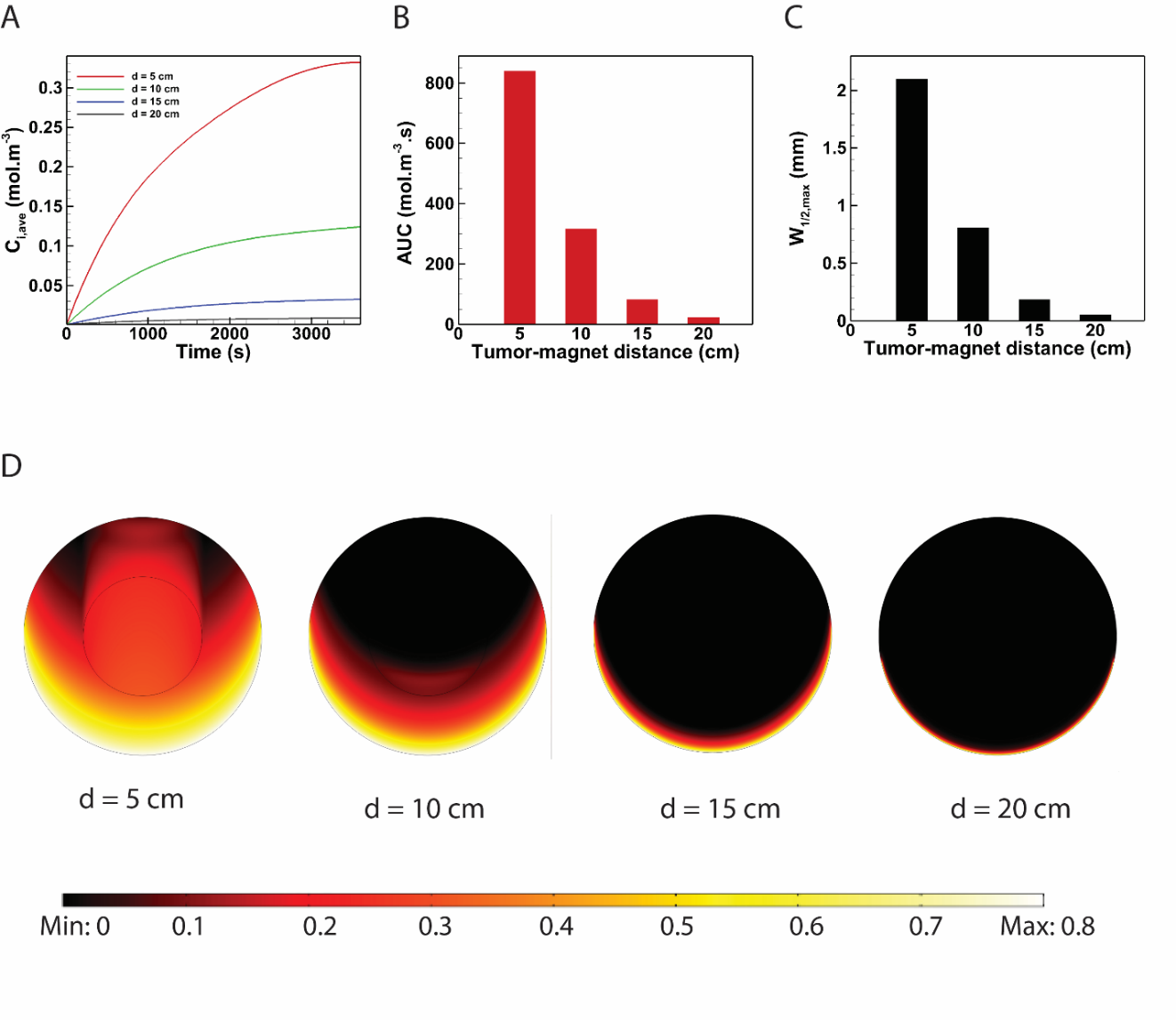** |
| --- |
| **Figure S3.** The effect of tumor-magnet distance on magnetically assisted IP drug delivery to a medium sized tumor nodule. (**A-C**) Compared to a large tumor (*R*=10 mm), the performance of MDT (characterized by parameters *C_i,ave_, AUC,* and *W_1/2,max_*) remains equally sensitive to the tumor-magnet distance in a medium sized tumor. *C_i,ave_, AUC*, and *W_1/2,max_* reduce by 36, 35, and 37 times, respectively as the tumor-magnet distance increases from 5 cm to 20 cm. (**D**) The intratumoral distribution of MNPs is conspicuously influenced by the tumor-magnet distance. |

| 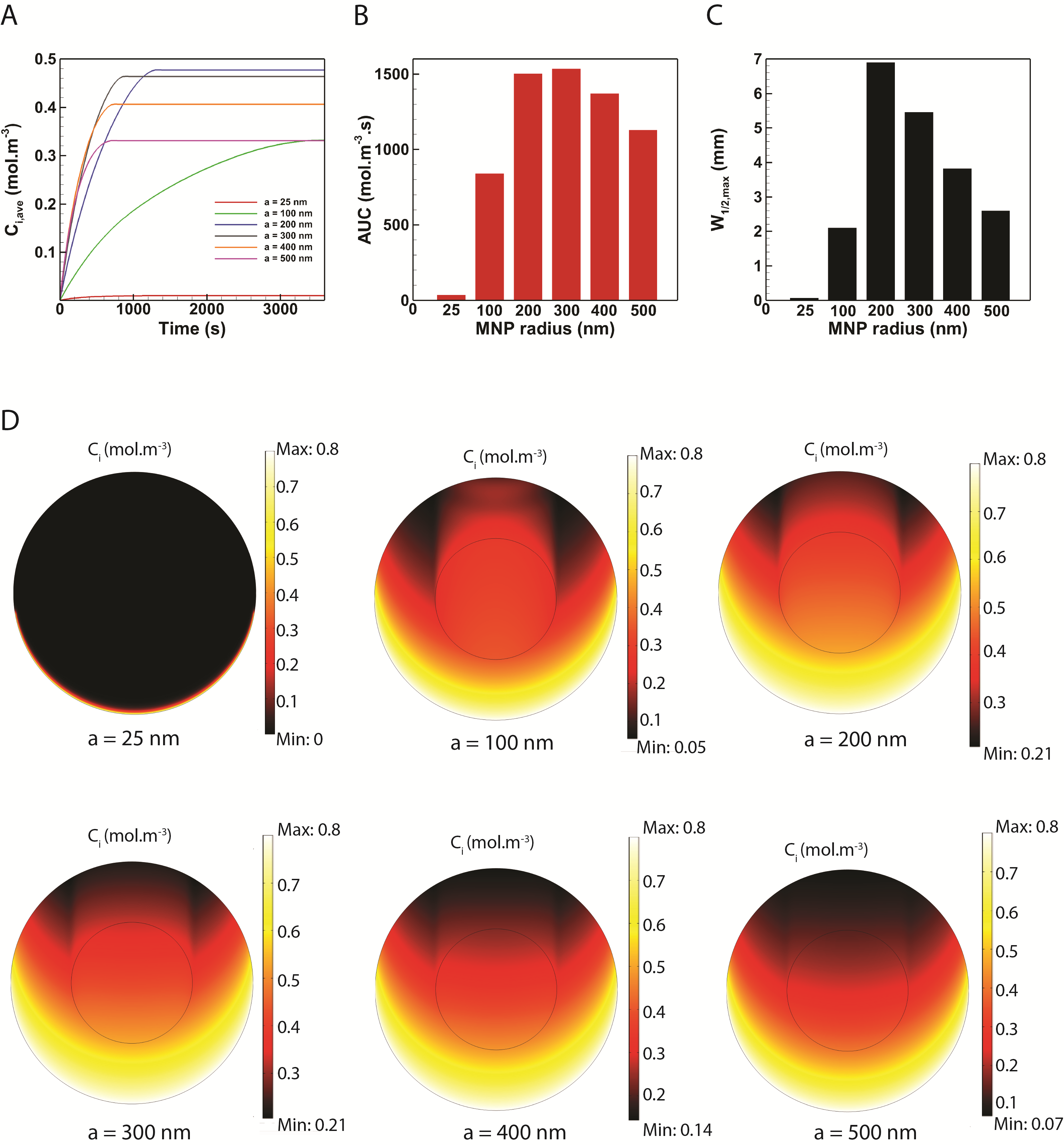 |
| --- |
| **Figure S4.** The effect of MNP size on magnetically assisted IP drug delivery to a medium sized tumor nodule. (**A-C**) The first two MDT performance parameters (*C_i,ave_* and *AUC*) are optimal for MNPs within the radius range of 200 nm-300 nm. The third MDT performance *W_1/2,max_* is, however, conspicuously maximal at a= 200 nm. Hence, the MDT performance is concluded to be optimal at a=200 nm. **(D**) MDT with particles of radii less than 25 nm is not efficacious in a small tumor nodule. Favorable intratumoral distributions of MNPs are achieved for a > 100 nm. |

**S2. Conventional and magnetically assisted IP chemotherapy for a small tumor (*R* = 1 mm)**

| 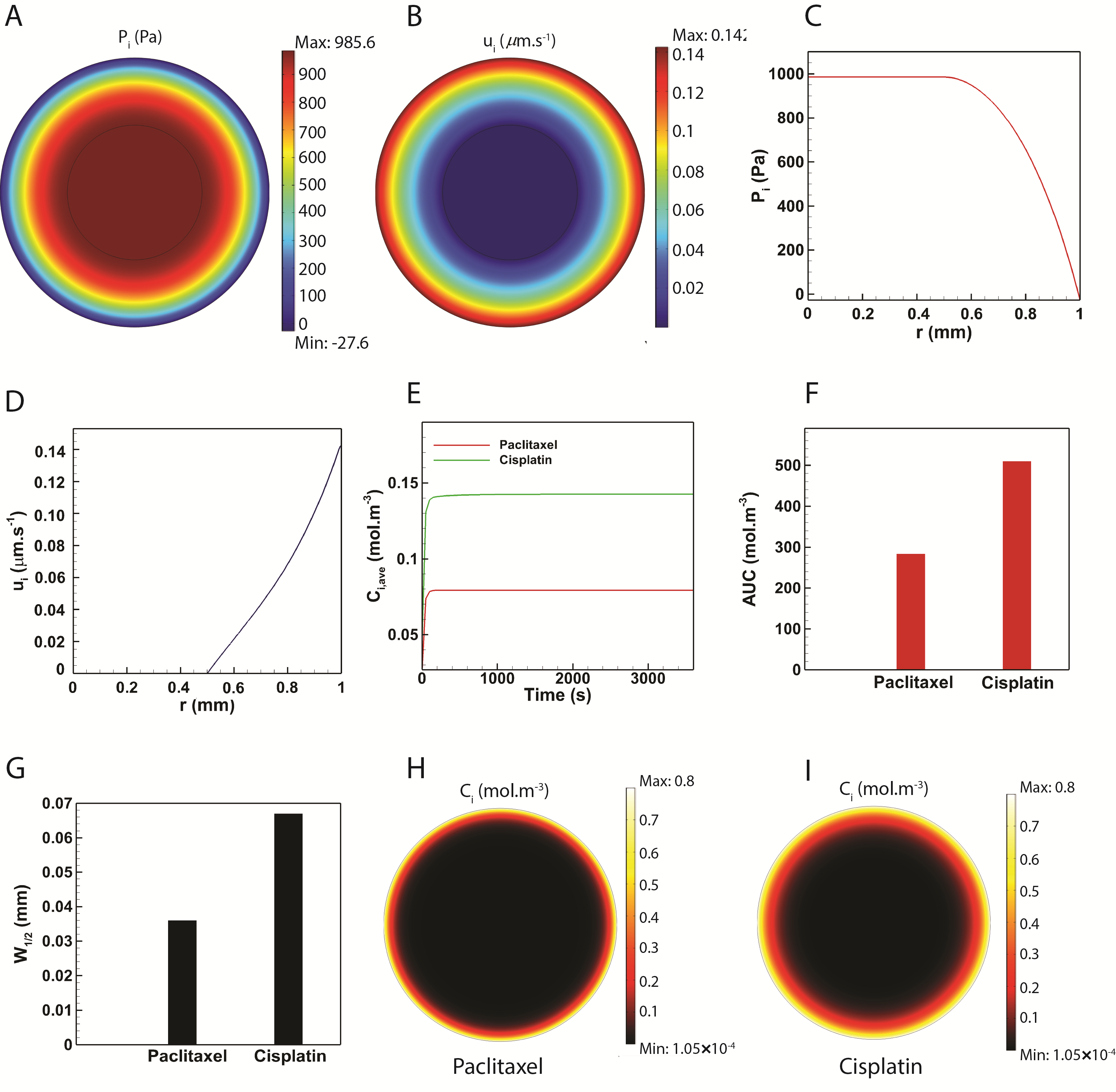 |
| --- |
| **Figure S5.** Conventional IP chemotherapy to a small tumor nodule. (**A-D**) Maximum values of IFP and IFV reduce by 36% and 18%, respectively, with respect to those of a large tumor (*R* = 10 mm). (**E-F**) The final intratumoral concentrations and the *AUC* of Paclitaxel and Cisplatin are almost 10 times greater than those of a large tumor (*R* = 10 mm) as a result of reduction in tumor size. (**G**) Even though the opposing IFV reduced with respect to a large tumor, the half width *W_1/2_* remains essentially the same since the diffusive transport of free cytotoxic agents is still negligible compared to opposing convective forces. (**H-I**) A larger percentage of the tumor bulk is covered with cytotoxic agents while the half width *W_1/2_* does not vary. This is obviously due to the smaller tumor size. |

| 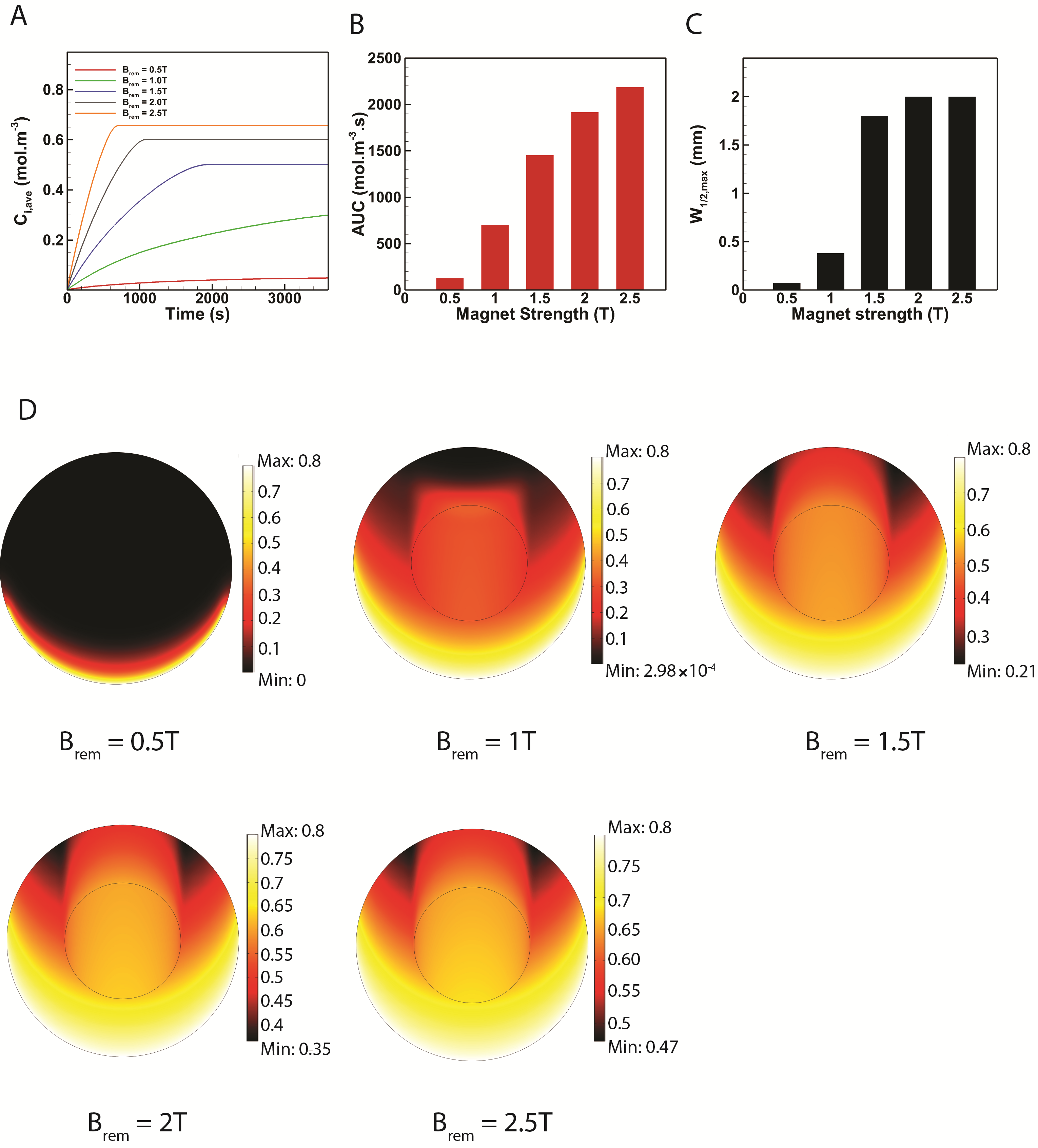 |
| --- |
| **Figure S6.** The effect of magnet strength on magnetically assisted IP drug delivery to a small tumor nodule. (**A-B**) The *AUC* and the value of *C_i,ave_* (at t= 1 hr) increases by 13 and 17 folds, respectively, as a result of a five-fold increase in the magnet strength from *B_rem_*= 0.5 T to *B_rem_*=2.5 T. It is concluded that the performance of MDT for a small tumor is less sensitive to the magnet strength compared to a medium sized (*R* = 5 mm) and a large (*R* = 10 mm) tumor. (**C**) The maximum half width *W_1/2,max_* is less sensitive to the choice of magnet strength when compared to larger tumors with 5 mm <*R* < 10 mm. (**D**) The intratumoral distribution of MNPs improves markedly by increasing the magnet strength from 0.5T to 1T. Nevertheless. further increasing the magnet strength has a little impact on intratumoral distribution of MNPs. |

| **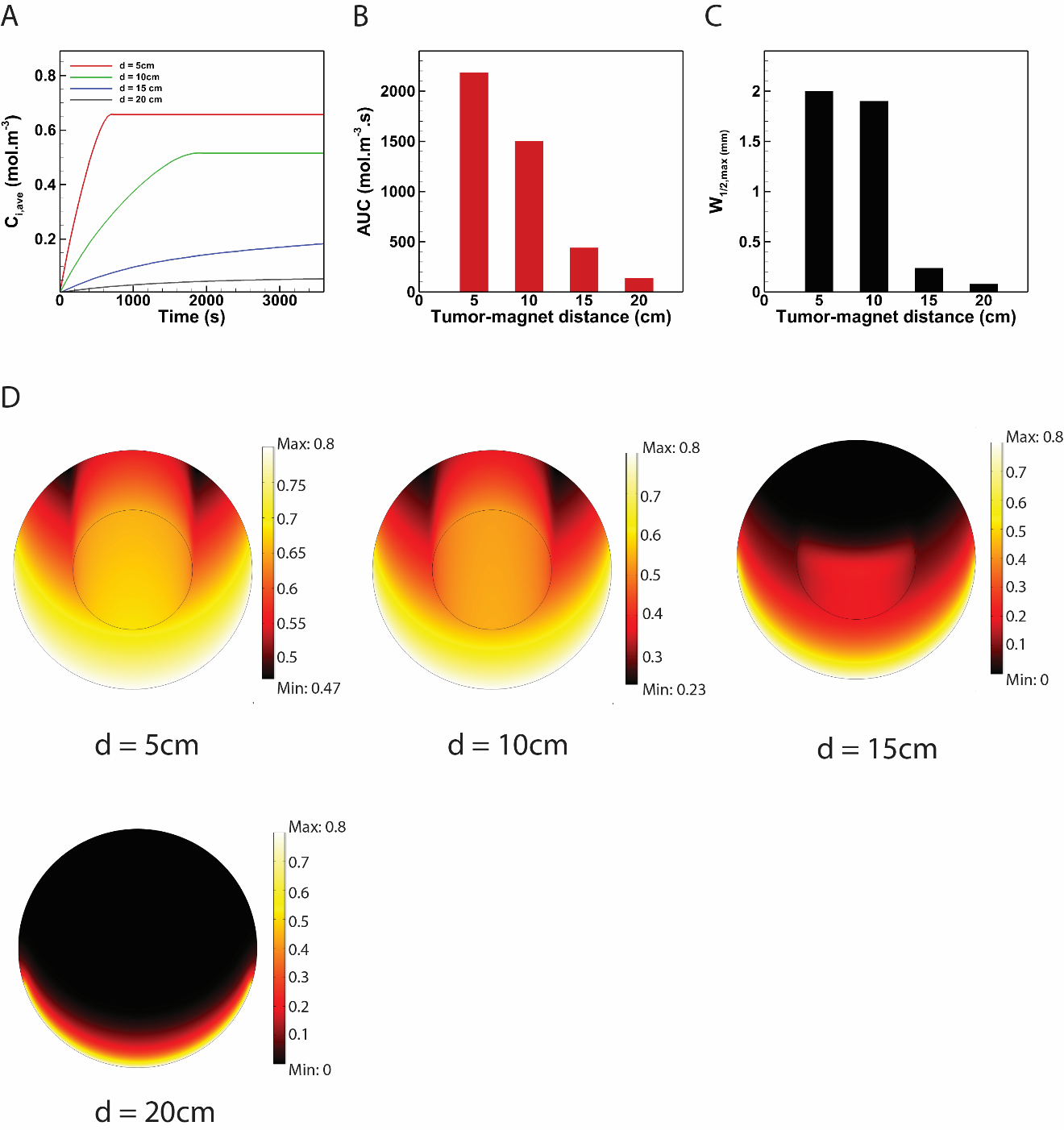** |
| --- |
|  |
| **Figure S7.** The effect of tumor-magnet distance on magnetically assisted IP drug delivery to a small tumor nodule. (**A-C**) For larger tumors (5 mm < R < 10 mm), acceptable MDT performance is obtained for larger tumor-magnet distances. For instance, *W_1/2,max_* drops by only 5% as the tumor magnet distance increases from 5 cm to 10 cm. The *W_1/2,max_* of a medium (*R* = 5 mm) and a large (*R* = 10 mm) tumor, however, drops by 61% and 63%, respectively, as the tumor magnet distance increases from 5 cm to 10 cm. (**D**) The intratumoral concentration of MNPs is minimally sensitive to tumor-magnet distance for 5 cm < d < 10 cm. |

| 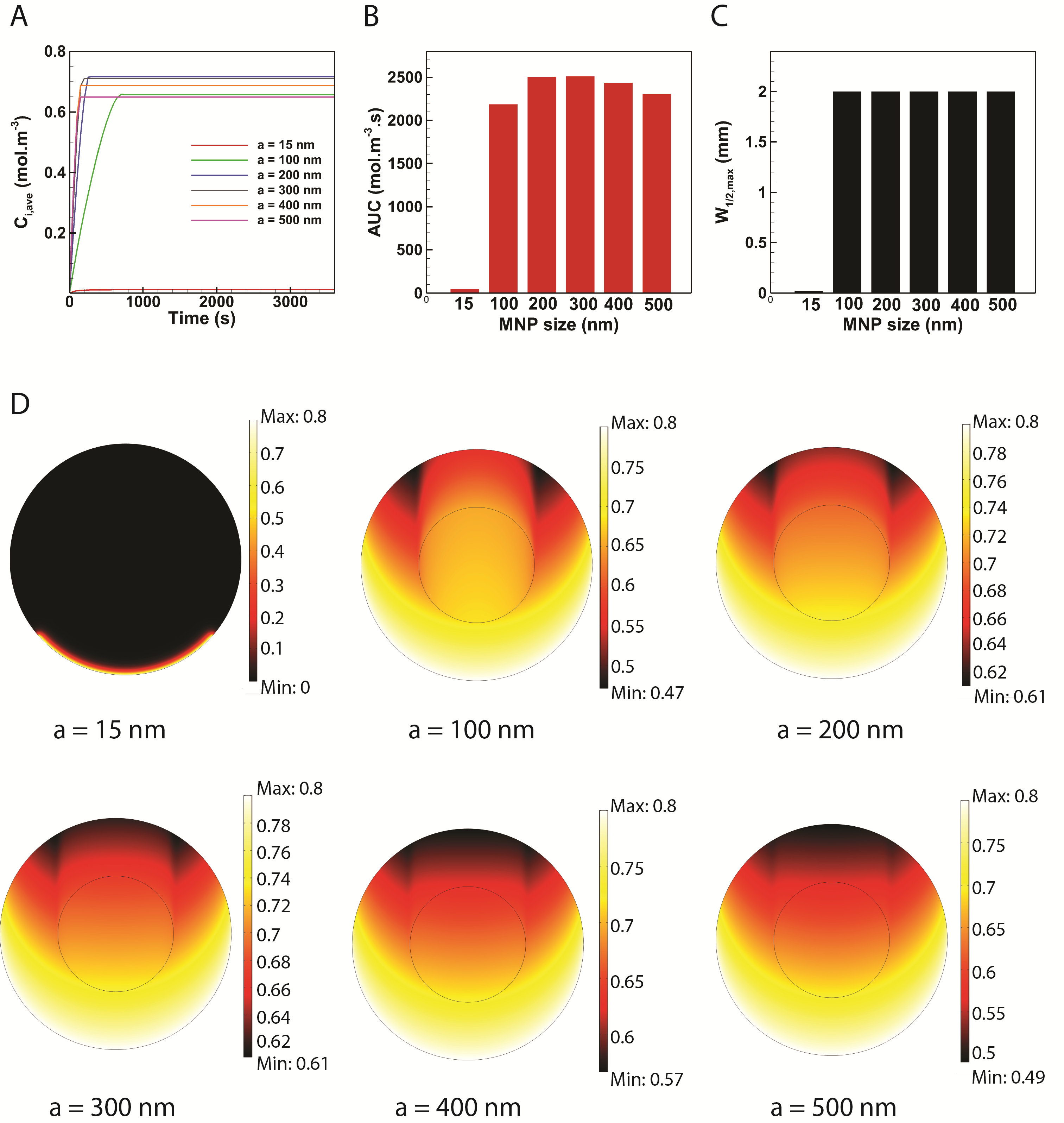 |
| --- |
| **Figure S8.** The effect of MNP size on magnetically assisted IP drug delivery to a small sized tumor nodule. (**A-C**) The MDT performance (described the values of *C_i,ave_, AUC*, and *W_1/2,max_*) minimally depends on the choice of MNP radius. **(D**) MDT with particles of radii less than 15 nm is not efficacious in a small tumor nodule. Favorable intratumoral distributions of MNPs are achieved for a > 100 nm. |
